# A Review of the Effects of Herbal Medicines on the Conductance of Ion Channels

**DOI:** 10.33549/physiolres.935720

**Published:** 2026-04-01

**Authors:** Mohammad Amin RAJIZADEH, Siyavash JOUKAR, Farzaneh ROSTAMZADEH, Maryam DOUSTAKI ZABOLI

**Affiliations:** 1 Physiology Research Center, Institute of Neuropharmacology, Kerman University of Medical Sciences, Kerman, Iran; 2 Cardiovascular Research Center, Institute of Basic and Clinical Physiology Sciences, Kerman University of Medical Sciences, Kerman, Iran; 3 Department of Physiology and Pharmacology, Afzalipour Medical Faculty, Kerman University of Medical Sciences, Kerman, Iran

**Keywords:** Plant derivatives, Ion channels, Therapeutic properties

## Abstract

Experimental evidence indicates that several phytochemicals can modulate ion channels currents, probably improving disorders linked to ion channels in cardiovascular, neurological, and gastrointestinal systems. This review critically evaluates the experimental evidence regarding the effects of bioactive phytochemicals from medicinal plants on ion channels properties. This review primarily focused on the beneficial effects of plant-derived bioactive agents on ion channels implicated in cardiovascular, neurological, and gastrointestinal disorders, specifically cardiac arrhythmias, epilepsy, anxiety, pain, and visceral smooth muscle dysfunction. Relevant literatures up to 2024 were gathered through comprehensive searches across multiple electronic databases. The results indicate that near 50 medicinal plants and their derivatives can modulate the conductivity of various Ca^2+^, K^+^, Na^+^, and Cl^−^ ion channels, alter the electrical properties of excitable cells, and affect the functional features of tissues and organ systems. These observations provide a foundational framework for researchers, health professionals and drug developers seeking to understand how botanical compounds impacts the functionality of ion channels.

## 1. Introduction

Ion channels are specialized transmembrane proteins for the passage of ions across biological membranes. These structures are typically depicted as narrow, aqueous pores that allow ions to pass through according to their size and electrical charge. This characteristic denotes the ability of the pore to discriminate between ions based on their physical and electrochemical properties [1]. The movement of calcium, potassium, and sodium ions is essential for various physiological functions such as electrical activity and contraction of striated, smooth, and heart muscles, insulin secretion, T-lymphocyte activation, and generation and propagation of neural signals. These ions can passively diffuse across the membrane through specific ion channels driven by their electrochemical gradients and membrane potential [2].

Ion channels, classified according to their ion selectivity and the stimuli that trigger their opening or closing, include voltage-gated sodium channels (Na_V_), voltage-gated calcium channels (Ca_V_), various voltage-gated potassium channels (K_V_) such as inward-rectifier potassium channel (K_ir_), certain chloride channels, and ligand-gated channels such as ATP-sensitive potassium channels (K_ATP_), acetylcholine-activated potassium channels (K_ACh_), small-conductance calcium-activated potassium channels (SK), transient receptor potential (TRP) channels, hyperpolarization-activated cyclic nucleotide-gated (HCN) channels, among others. Some receptors, such as ryanodine receptors (RyR) and inositol 1,4,5-trisphosphate (IP_3_) receptors, which are located in the membrane of the endoplasmic reticulum, mediate the release of calcium from intracellular stores into the cytoplasm [3–6]. Table 1 summarizes various types of ions channels and their localization, distribution, common inhibitors, and key function [7–9].

Each ion channel plays a specific role in regulating the membrane potential and synergistically contributes to the generation of action potential in excitable cells. Dysfunction of these channels, resulting from genetic mutations or post-translational modifications, can disturb cell function, leading to various diseases, such as myocardial hypertrophy, arrhythmias, heart failure, epilepsy, ileus, and other channelopathies [10].

Many synthetic drugs can treat conditions like convulsion and cardiac arrhythmia by modulating the function of ion channels [11]. However, the same mechanism of action can also cause serious side effects such as dangerous arrhythmias and instability in the nervous system, thereby increasing the risk of death.

**Fig. 1:**
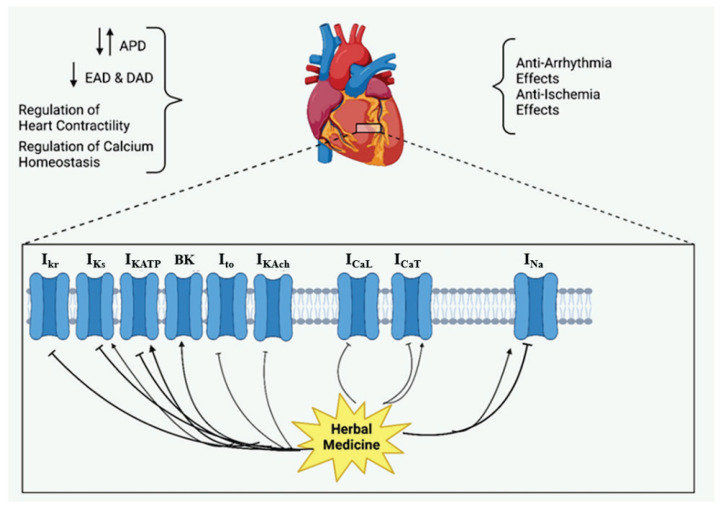
The effects of herbal medicine or their components on cardiac ion channels. Arrow means stimulation, brake means inhibition**.** APD – action potential duration, EAD – early after-depolarization, DAD – delayed after-depolarization.

**Fig. 2:**
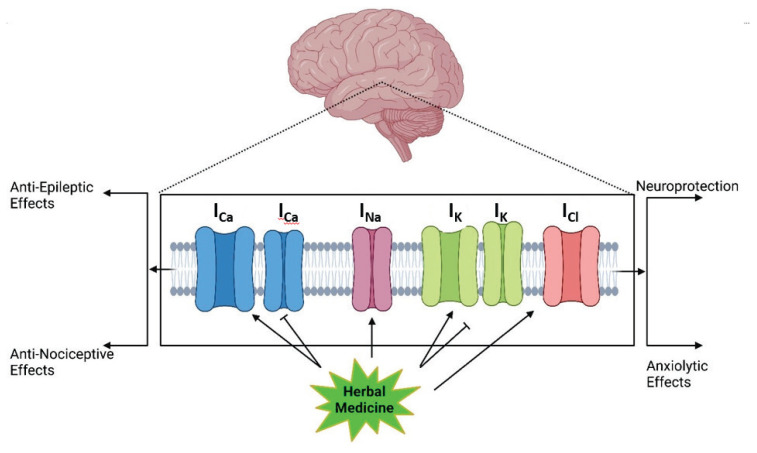
The effects of herbal medicine or their components on brain ion channels. Arrow means stimulation, brake means inhibition.

**Fig. 3:**
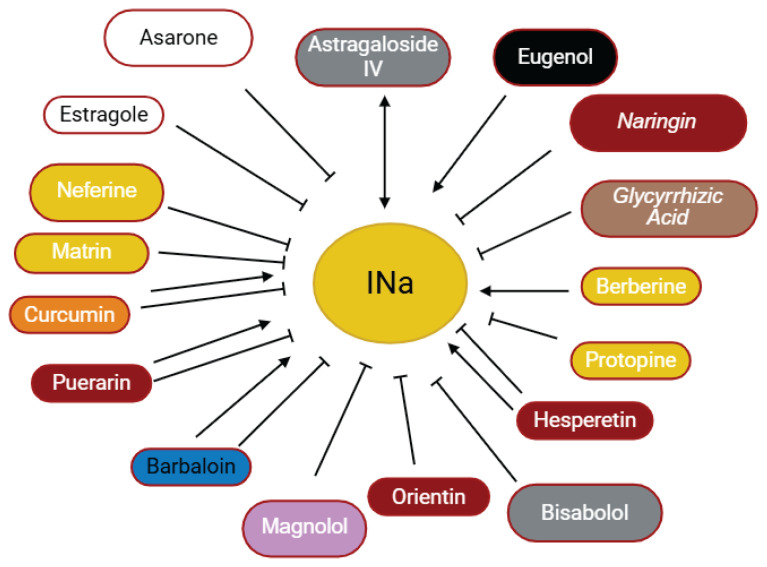
The effects of some herbal medicine or their components on sodium current. Arrow means stimulation, brake means inhibition. Yellow boxes: Alkaloids, Red boxes: Flavonoids, Black box: Benzene, Blue box: Quinone, Gray boxes: Terpenoids, Orange boxes: Polyphenols, Brown box: Saponin, Pink box: Lignan, White boxes: Phenyl propene.

**Fig. 4:**
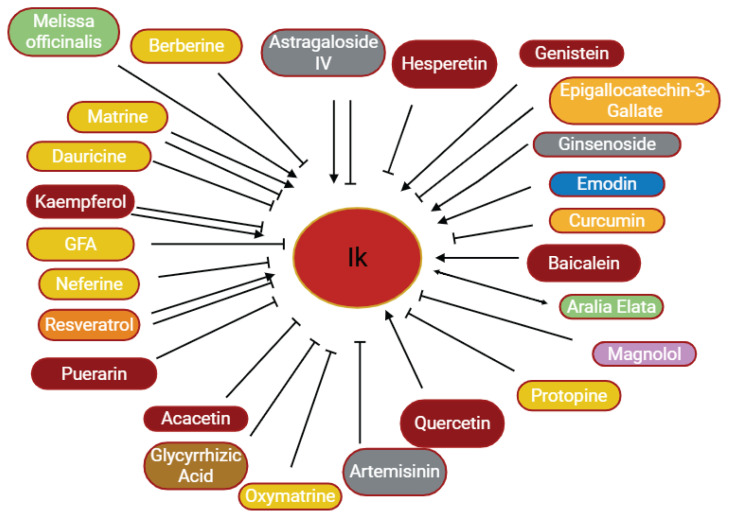
The effects of some herbal medicine or their components on potassium current. Arrow means stimulation, brake means inhibition. Yellow boxes: Alkaloids, Red boxes: Flavonoids, Green boxes: Plants, Blue box: Quinone, Gray boxes: Terpenoids, Orange boxes: Polyphenols, Brown box: Saponin, Pink box: Lignan.

**Fig. 5:**
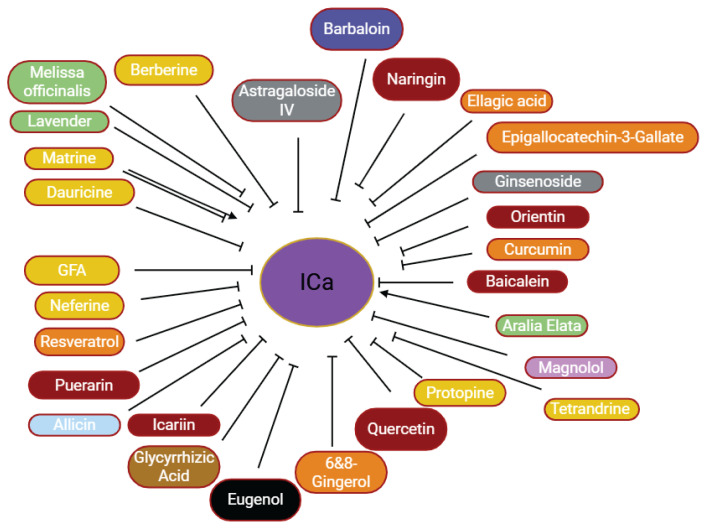
The effects of some herbal medicine or their components on calcium current. Arrow means stimulation, brake means inhibition. Yellow boxes: Alkaloids, Red boxes: Flavonoids, Green boxes: Plants, Blue box: Quinone, Gray boxes: Terpenoids, Orange boxes: Polyphenols, Brown box: Saponin, Pink box: Lignan, Black box: Benzene, Light blue box: Sulfur compounds.

**Fig. 6:**
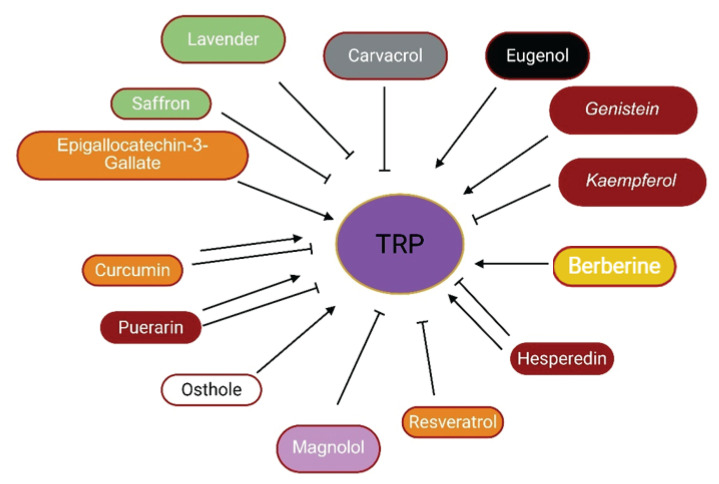
The effects of some herbal medicine or herbal components on TRP channels. Arrow means stimulation, brake means inhibition. Red boxes: Flavonoids, Black box: Benzene, Gray box: Terpenoids, Orange boxes: Polyphenols, Pink box: Lignan, White box: Coumarin, Green boxes: plants, yellow box: Alkaloid.

For thousands of years, plants have been recognized as a rich reservoir of biologically active substances for therapeutic use [12]. Their widespread use in traditional medicine is attributed to their affordability and accessibility [13]. The basic mechanisms of action of some herbal medicines are increasingly being covered, revealing that many plants and their constituents exert therapeutic and/or side effects through the modulation of specific ion channels. Given the limited number of reviews on this subject, this narrative review aimed to consolidate current knowledge by focusing on the effects of herbal medicines on various ion channels across different body systems. We also described the modulatory mechanisms of specific herbal compounds, with supplementary data summarized in Tables 2–4 as well as in Figures 1–6.

## 2. Search Methods

A comprehensive and systematic search was conducted across seven major scientific databases: Scopus, Springer Link, EMBASE, Science Direct, PubMed, Google Scholar, and Web of Science. The search strategy incorporated the following key terms: ion channel modulation, plant-derived ion channel modulators, phototherapeutic compounds, bioactive plant constituents, secondary plant metabolites, and traditional medicinal plants. Publications up to November 2024 were considered. Studies were eligible for inclusion if they involved preclinical investigations and clinical trials. Botanical substances exhibiting cardiac rhythm stabilization, analgesic, neurological effects, particularly anticonvulsant actions, smooth muscle relaxation, or cholinergic pathway modulation were selected. The selection process followed a rigorous two-phase screening approach, beginning with title and abstract assessment, followed by full-text evaluation of potentially relevant studies. To ensure methodological consistency, the search strategy was guided by the Population, Intervention, Comparator, and Outcome (PICO) framework. To maintain conciseness without compromising completeness, when multiple studies on the same botanical agent reported comparable findings, preference was given to the most recent or the highest-quality study. To minimizes the selection bias, two independent reviewers performed the screening. Any disagreements were resolved through consensus discussion, thereby enhancing the reliability and validity of the included studies.

## 3. Results

### 3.1. Plants

#### 3.1.1. *Aralia elata*

The Japanese, Chinese, or Korean angelica tree (*Aralia elata*) is a woody plant species in the family *Araliaceae* [14]. The positive inotropic effect of *Aralia elata* observed in canine myocardium and isolated rat cardiomyocytes is potentially mediated by an increase in the amplitude of the cytosolic calcium transient [22]. It has been also shown to boost the amplitude of L-type calcium channels (LTCC) current in heart of rats with diabetic cardiomyopathy [21], identifying a likely pathway for the elevated intracellular calcium.

#### 3.1.2. Lavender

*Lavandula angustifolia* is an evergreen plant native to the Mediterranean, known for its fragrant flowers and essential oil, which are also used medicinally. Lavender oil has calming effects and may help relax certain muscles. It also shows antibacterial and antifungal properties [15]. Lavender has been shown to modulate transient calcium channel (T-type Ca^2+^ channels), contributing to its analgesic and sedative effects [16]. It also inhibits N- and P/Q-types voltage-gated calcium channels in the hippocampus [17,18]. Hashimoto *et al.* [19] further revealed inhibitory effects of linalool, an essential component of lavender oil on nociceptive transient receptor potential cation channel A1 (TRPA1) and voltage-gated Ca^2+^ channels in mouse sensory neurons.

#### 3.1.3. *Melissa officinalis* L

*Melissa officinalis* is an aromatic and medicinal plant commonly known as lemon balm, honey balm, or bee balm. *Melissa officinalis* is an excellent source of terpene and polyphenolic compounds, including phenolic acids such as rosmarinus acid, caffeic acid, and protocatechuic acid; flavonoids such as quercitrin, rhamnocitrin, luteolin; and tannins [20]. Lemon balm has sedative and mild hypnotic, hypoglycemic, hepatoprotective, antibacterial, anti-inflammatory, antioxidant, antiviral, antispasmodic, anxiolytic, neuroprotective, cardioprotective, and antiarrhythmic properties [20–22].

The mechanisms underlying the anxiolytic effects of *Melissa officinalis* remain controversial. Some studies reported that *Melissa officinalis* activates the GABAergic system and increases chloride ion conductance, whereas others suggested that its effects are mediated through modulation of the cholinergic system and voltage-dependent calcium channels [23]. Gazola *et al.* [24] demonstrated that *Melissa officinalis* may reduce heart rate *via* stimulation of cardiac muscarinic receptors or inhibition of voltage-dependent calcium channels. The antiarrhythmic effects of this plant have also been attributed to the inhibition of calcium channels and the activation of voltage-dependent potassium channels [22,25].

#### 3.1.4. *Panax notoginseng* saponins

The roots of *Panax notoginseng* (Burk.) are used as a source of *Panax notoginseng* saponins*,* which have been shown to exhibit antiarrhythmic effects [26]. *Panax notoginseng* saponins modulates potassium and calcium ion channels, reducing cardiomyocyte automaticity, slowing cardiac electrical conduction, prolonging action potential duration and effective refractory period, and preventing reentry arrhythmias [27]. Ginsenoside Rg1, as a monomer of *Panax notoginseng saponins*, has been reported to prolong sinus node recovery time, atrioventricular conduction Wenckebach cycle length, and ventricular effective refractory period, as well as to increase the ventricular fibrillation threshold, producing cardiac electrophysiological effects similar to those of amiodarone [28].

#### 3.1.5. Saffron

Saffron, *Crocus sativus* L., is a widely used spice with numerous documented pharmacological properties, including neuroprotective, cardiovascular protective, antinociceptive, antiarrhythmic, anxiolytic and bronchodilator effects [29–31]. Its major bioactive constituents are crocin, crocetin, picrocrocin, and safranal. Electrophysiological studies using the patch-clamp technique have shown that crocetin and safranal significantly inhibit the human ether-a-go-go-related gene (hERG) K^+^ current, whereas crocin and picrocrocin do not exert such effects [32]. hERG is coded by KCNH2 gene, which is the pore-forming subunit responsible for rapid delayed-rectifier potassium current (I_Kr_). Crocetin has been reported to inhibit LTCC in adult rat ventricular myocytes, leading to reduced intracellular Ca^2+^ levels and decreased contractility [33]. Safranal has antinociceptive activity through partial agonism and selective desensitization of TRPA1 [34]. It also induces vasodilatation by inhibiting calcium influx through LTCC and suppressing the Na^+^/Ca^2+^ exchanger [35]. Saffron exert antiarrhythmic effect at least in part through inhibition of calcium channels [36].

#### 3.1.6. *Salvia miltiorrhiza*

*Salvia miltiorrhiza* Bunge, a traditional Chinese medicines, is officially listed in the Chinese Pharmacopoeia [37]. The dried roots of *Salvia miltiorrhiza* Bunge are widely used for their therapeutic effects, including promoting blood flow, removing blood stasis, alleviating intestinal gurgling, dissolving calculi, relieving abdominal fullness, and reducing swelling [38]. The mechanism underlying *Salvia miltiorrhiza-*induced contraction remains uncertain; however, it appears not to be mediated by muscarinic receptors, sodium channels, or calcium channels, but rather through the increase in cytosolic Ca^2+^ concentration and the activation of the Ca^2+^-calmodulin pathway [39]. It also has antiepileptic effects through increasing presynaptic Ca^2+^ influx [40].

#### 3.1.7. Turmeric

Turmeric, *Curcuma longa* L., a member of the *Zingiberaceae* family, is a widely used culinary spice with numerous pharmacological properties. Curcumin, its principal bioactive constituent, has neuroprotective, antioxidant, anticancer, anti-inflammatory, hepato-protective, immunomodulatory, cardioprotective, antide-pressant, antifertility, antiallergic, antimicrobial, and antidermatophytic properties [41]. Curcumin suppresses early and delayed afterdepolarization like arrhythmogenic activities, shortens action potential duration, and inhibits late sodium current (I_NaL_), transient sodium current (I_NaT_), L-type calcium current (I_CaL_), and I_Kr_ [42]. Curcumin also show the antiepileptic (anticonvulsant) effects [43], which may be mediated by modulation of neuronal sodium channels and downregulation of Na_v_1.1 expression in the cortex [43,44]. Evidence from animal studies suggests that its analgesic effects involve activation of both cannabinoid and opioid systems, potentially through the release of endogenous endocannabinoids and opioids [45]. Curcumin inhibits glutamate release from rat prefrontocortical synaptosomes by suppressing presynaptic voltage-gated calcium channels of N- and P/Q-type [46]. Curcumin appears to mediate antidepressant-like effects through ion channel modulation. including inhibition of Ca^2+^ release-activated Ca^2+^ (CRAC) channels [47]. It also reduces Kv currents in rabbit coronary arterial smooth muscle cells [48]. Additionally, curcumin alleviate diabetic neuropathic pain by modulating voltage-gated sodium channels and increasing sodium levels in dorsal root ganglion neurons [49]. Curcumin also exhibits gastroprotective effects *via* activation of the nitric oxide (NO) / cyclic guanosine monophosphate (cGMP) / K_ATP_ channel pathway [50]. Furthermore, it functions as a multi-ion channel blocker, with a preferential inhibitory effect on the late sodium current, suggesting potential antiarrhythmic benefits [42]. In addition, curcumin has been shown to have inhibitory, excitatory, and modulatory effects on TRP channels [51].

#### 3.1.8. Salvianic acid A

Danshensu, dried root of *Salvia miltiorrhiza,* is widely used in traditional medicine for the treatment of coronary artery disease [52]. Salvianic acid A reduce the myocardial ischemia injury possibly by preventing I_CaL_ current and decreasing myocardial contractility [53].

### 3.2. Plant derivatives

#### 3.2.1. 6-gingerol

6-gingerol, one of the most abundant and bioactive constituents of *Zingiber officinale* Roscoe, ginger, exhibits anticancer, anti-inflammatory, antioxidant and cardiometabolic protective effects [54]. Using patch-clamp technique and Ion Optix system, it has been demonstrated that 6-gingerol inhibits L-type calcium channels at a concentration-dependent manner in ischemic cardio-myocytes, reducing Ca^2+^ influx, stabilizing intracellular Ca^2+^ homeostasis, attenuating oxidative stress, and ultimately decreasing myocardial infarct size, and improving cardiac function [55].

*Zingiber officinale* and its active components have analgesic effect through serotonergic pathways, nitric oxide (NO) signaling, K_ATP_ channel, and modulation calcitonin gene related peptide (CGRP) [56]. 6-gingerol acts as an anticonvulsant by interacting with the amino-terminal domain, glutamate-binding site, and ion channel of the NR2B containing N-methyl-D-aspartate (NMDA) receptor, thereby restoring the balance between GABAergic and glutamatergic in the epileptic brain [57].

#### 3.2.2. 8-gingerol

8-gingerol has been reported to exert antioxi-dant, anti-inflammatory, and cardiovascular protective effect [58]. 8-gingerol regulates the mitogen-activated protein kinase (MAPK) signaling pathway, inhibits LTCCs, decreases calcium overload, alleviates oxidative stress damage, and prevents cardiomyocyte apoptosis. These actions collectively contribute to its protective effects against myocardial ischemic injury [59].

#### 3.2.3. Acacetin

The bioactive flavonoid acacetin, isolated from *Saussurea involucrata* exhibits considerable antiarrhythmic effects [60]. Pharmacological studies indicate that its mechanism involves the selective blockade of K^+^ channels, with negligible effects on Na^+^ channels or LTCC. A study in a controlled canine model also demonstrated that acacetin inhibited experimentally induced atrial fibrillation. This effect occurred without QT interval prolongation, suggesting a low risk for pro-arrhythmic side effects and a desirable safety profile [61].

#### 3.2.4. Aconitine

Aconitine is a C19 norterpenoid alkaloid produced by some medicinal plants such as *Aconitum carmichaelii Debx*. Although it has potential applications to treat cancer, pain, inflammation and immune related diseases [62], its use is limited by a considerable toxicity. By binding to voltage-gated sodium channels, aconitine like mesaconitine induces the persistent activation of sodium channels, preventing its normal inactivation and becoming refractory to next excitation. The consequent uncontrolled sodium influx results in toxic effects on the nervous system, heart, and muscles. Because of this specific cardiotoxicity, aconitine is used for the arrhythmia induction in experimental studies [63,64].

#### 3.2.5. Allicin

Allitridum (Allicin), the primary bioactive compound found in garlic (*Allium* genus*, Liliaceae* family), exhibits significant therapeutic potential in managing and preventing cardiovascular diseases [65]. The specific mutation, lysine-proline-glutamine deletion (ΔKPQ), in sodium voltage-gated channel alpha subunit 5 (SCN5A) causes a well-known long QT syndrome type 3 (LQT3), an inherited cardiac arrhythmia disorder. A study showed that allicin decreases the late sodium current associated with the ΔKPQ-SCN5A mutation, likely by enhancing steady-state and intermediate-state inactivation of the channel, thereby reducing persistent leak of sodium ions [65]. It has been also shown that in rat cerebrocortical nerve terminals, allicin depresses glutamate release and decreases N- and P/Q-type channel activity and suppresses Ca^2+^ influx and protein kinase C (PKC) activity [66]. Furthermore, allicin also attenuates transient outward potassium currents in mouse ventricular myocytes [67].

#### 3.2.6. Aloperine

Aloperine, quinolizidine alkaloid isolated from *Sophora alopecuroides* L., exhibits potential therapeutic effects in cardiovascular diseases including hypertension, ventricular remodeling, myocardial ischemia, and arrhythmias [68]. Studies indicate that aloperine targets key cardiac ion channels. At a concentration of 100 μM, it did not affect cloned K_v_4.3 channels but increased the I_Kr_ current [68,69]. Furthermore, in rat ventricular myocytes, aloperine significantly reduced the peak density of voltage-gated sodium current in a concentration-dependent manner [70].

#### 3.2.7. Artemisinin

Artemisinin, a compound derived from the Qinghao plant, *Artemisia annua* L., has been suggested as an antiarrhythmic substance. In a study artemisinin attenuates I_Na_ by modulating the voltage dependence of the Na^+^ channel similar to the class I antiarrhythmic agents [71].

#### 3.2.8. Asarone

Asarone isomers are phenylpropene secondary metabolites naturally occurring in species such as *Acorus calamus* L., *Guatteria gaumeri* Greenman and *Aniba hostmanniana* Nees. Chemically, they are classified into propenylic isomers (trans-α and cis-β asarone) and an allylic isomer (γ-asarone) [72]. The antiepileptic mechanism of these compounds is mediated through the inhibition of voltage-gated calcium and sodium channels [73].

#### 3.2.9. Astragaloside IV

Astragaloside IV is a saponin purified from *Astragalus membranous* (Fisch) Bunge, which is widely used in the treatment of diabetes. It has been reported to possess various anti-inflammatory, antioxidant, antiviral and immunomodulatory effects [74]. Astragaloside IV prolongs the action potential duration in guinea pig ventricular myocytes, which can be attributed to its inhibitory effect on the K^+^ current. In addition, astragaloside IV suppresses I_CaL_ to affect Ca^2+^ signaling [74].

#### 3.2.10. Baicalein

Baicalein is the principal active ingredient of *Scutellaria baicalensis Georgi*. Several preclinical studies reported cardioprotective effects of baicalein. However, its low oral bioavailability greatly limits clinical efficacy [75]. It has been shown baicalein can alleviate myocardial ischemic injury by inhibiting I_CaL_ and reducing intracellular calcium level [75].

#### 3.2.11. Barbaloin

*Aloe vera* contains the bioactive compound barbaloin, which exhibits anti-inflammatory, antiarrhythmic, antitumor and antibacterial properties [76]. In rabbit ventricular myocytes, barbaloin has been shown to eliminate early and delayed after-depolarization [77]. Moreover, barbaloin inhibits I_CaL_ and peak sodium current in a dose-dependent manner, thereby reduces ventricular arrhythmias [78].

#### 3.2.12. Berberine

Berberine is an isoquinoline alkaloid derived from the roots, stems, and bark of plants including barberry, Chinese goldthread, goldenseal, tree turmeric, and Oregon grape. Extracts from berberis have been reported to exhibit antihypertensive and antiarrhythmic effects [79–81]. Antiarrhythmic and anticonvulsant effects of those plants have also been attributed to berberine [82,83]. Berberine prolongs the action potential duration and the effective refractory period of myocardial cells, thereby inhibiting the occurrence of atrioventricular reentrant tachycardia through the suppression of potassium and calcium ion channels [82] and HCN cation channel [84,85]. In a rat model, berberine alleviate cisplatin-induced peripheral neuropathy by modulating inflammation signal *via* TRPV1[86].

#### 3.2.13. Carvacrol

Carvacrol, a natural monocyclic monoterpenoid (2-methyl-5-isopropyl phenol) and a major component of thyme oil, possesses several biological activities, including antioxidant properties [87,88]. It has been shown that carvacrol can reduce the action potential upstroke velocity, action potential duration and conductive velocity in heart of rabbit and human by inhibiting Ca^2+^and Na^+^ current and blocking transient receptor potential cation channel subfamily M member 7 (TRPM7) channels [87]. TRPM7 belongs to the TRP family of ion channels, mainly conducts divalent cations such as Mg^2+^, Ca^2+^, and Zn^2+^ (Table 1).

#### 3.2.14. Dauricine

Dauricine, a bisbenzyltetrahydroisoquinoline alkaloid extracted from the roots of *Menispermum dauricum DC*, exhibits notable antiarrhythmic effects [89]. Dauricine prolongs the atrial effective refractory period, action potential duration, and significantly blocks Na^+^, K^+^, and Ca^2+^ currents in myocardial tissues, thereby demonstrating its antiarrhythmic properties [90].

#### 3.2.15. Ellagic acid

Ellagic acid, a natural polyphenolic component present in berries, has been demonstrated to exhibit antioxidant, anticancer and anti-inflammatory properties [91]. Ellagic acid can affect ionic and mechanical properties of rat ventricular myocytes. Ellagic acid through the activation of nitric oxide synthase - guanylate cyclase - cGMP pathways lead to reduction of I_CaL_ and results in negative inotropic effects. Therefore, ellagic acid may be useful in the pathophysiological conditions such as hypertension and coronary artery diseases [91].

#### 3.2.16. Elatoside C

Elatoside C, a natural saponin isolated from *Aralia chinensis* L., has antioxidative activity [92]. The elatoside C has been shown to protect the heart against ischemia/reperfusion injury (I/R) by modulating intracellular Ca^2+^ homeostasis through regulation of calcium channels. Specifically, elatoside C improves abnormal calcium handling during I/R injury by enhancing the activity of sarcoplasmic reticulum Ca^2+^-ATPase (SERCA2), reducing endoplasmic reticulum (ER) Ca^2+^ depletion, and decreasing the expression of ER stress protein markers [93].

#### 3.2.17. Emodin

Emodin, a natural anthraquinone derivative extracted from traditional medicinal herbs such as *Rheum officinale* and *Polygonum cuspidatum*, exhibits diverse pharmacological properties, including anticancer, anti-inflammatory, antioxidant, and antibacterial effects [94]. Moreover, the vasorelaxant effect of emodin is mediated by the activation of large-conductance calcium- and voltage-activated potassium (BK) channels [95].

#### 3.2.18. Epigallocatechin-3-gallate

Epigallocatechin-3-gallate, a polyphenol abundant in green and white tea and present at lower concentrations in black tea, has been reported to exert protective effects against heart injury and related cardiovascular pathology [96–98]. In neonatal rat ventricular myocytes, epigallocatechin-3-gallate has been shown to activate the sodium current. At a concentration of 10 μM, it slows down the inactivation and accelerates the recovery from inactivation [99]. Epigallocatechin-3-gallate has been found to inhibit the I_CaL_ and I_Kr_, slow delayed-rectifier potassium current (IKs), transient outward potassium channel (Ito), and KATP currents, leading to a decrease in the action potential duration [100,101]. It also interacts with the TRPA1 in several physiological systems. In the cardiovascular system, epigallocatechin-3-gallate activates TRPA1 on perivascular sensory nerves, promoting calcium influx and the release of CGRP, which leads to neurogenic vasodilation and reductions in blood pressure [102]. Within the nervous system, oxidized epigallocatechin-3-gallate derivatives activate TRPA1 in dorsal root ganglion neurons, driving Ca^2+^ entry and sensory-neuropeptide release, indicating a role in nociceptive signaling [103].

#### 3.2.19. Eugenol

Clove and basil essential oils contain eugenol, a phenylpropene compound obtained through fractionation [104]. Eugenol has been shown to modulate the excitability of rat sciatic nerve and superior cervical ganglion neurons by inhibiting Na^+^ channels [104]. Eugenol increases intracellular Ca^2+^ levels by activating TRPA1 that may contribute to the desensitization of pain sensation and analgesic effects in trigeminal ganglion neurons [105,106].

#### 3.2.20. Genistein

Genistein, an isoflavone primarily extracted from soybeans, is a well-known phytoestrogen with various biological activities, including antiproliferative, anti-inflammatory, and antioxidant effects [107]. Through a protein tyrosine kinase-dependent mechanism, genistein has been shown to inhibit BK channels, suppress cell cycle progression, and ultimately reduce low-density lipoprotein (LDL)-induced proliferation of vascular smooth muscle cells [107]. Genistein has been found to amplify TRPC5-mediated Ca^2+^ influx in both TRPC5-overexpressing HEK cells and primary bovine aortic endothelial cells [108].

#### 3.2.21. Ginsenoside

Ginsenosides, the active components of the natural medicinal herb ginseng*,* exhibit antiarrhythmic effects by inhibiting Ca^2+^ channels and modulating intracellular Ca^2+^ signaling [109]. Specific ginsenosides such as Re, Rb1, and Rg2 have been shown to block LTCC [110]. Using the patch-clamp technique, it was demonstrated that ginsenoside Re increases I_Ks_, reduces I_CaL_ and Ca^2+^ overload, and protects the heart from I/R injury, through the cGMP-dependent pathway [111]. In rabbit ventricular myocytes subjected to I/R injury, ginsenoside Rb1 was found to block both I_NaL_ and I_CaL_ currents, decrease action potential amplitude, prevent delayed afterdepolarizations induced by elevated calcium, and protect cardiomyocytes from calcium overload [112]. Furthermore, ginsenosides have been reported to exhibit antiepileptic effects by reducing calcium influx [113].

#### 3.2.22. Glycyrrhizic acid

Licorice, derived from the root and rhizome of *Glycyrrhiza uralensis* Fisch., *Glycyrrhiza inflata* Bat., or *Glycyrrhiza glabra* L., contains glycyrrhizic acid as its primary active component [114]. The antiarrhythmic effects of glycyrrhizic acid are attributed to its ability to block sodium and calcium ion channels, to inhibit pacemaker cell automaticity, to slow conduction speed, and to prolong action potential duration and the effective refractory period [115].

#### 3.2.23. Guanfu base A

The roots of *Aconitum coreanum* are used to extract guanfu base A, an alkaloid from the *Ranunculaceae* family with antiarrhythmic effects [116]. Guanfu base A acts primarily as a multichannel blocker, including inhibition of sodium channels. It reduces action potential amplitude and V_max_, and prolongs action potential duration and effective refractory period, converts unidirectional conduction block to bidirectional conduction block, and decreases the incidence of premature contraction and atrioventricular reentry [117]. It has been shown that guanfu base A has antiarrhythmic effects through inhibiting I_Ks_ and I_Ca_ in the heart [118].

#### 3.2.24. Hesperidin and Hesperetin

Hesperidin and its aglycone, hesperetin, are flavanones primarily found in the young fruit of *Citrus* species (*Rutaceae*) and are increasingly consumed in Western diets [119]. Hesperetin is a potent blocker of Na^+^ channels, binding to these channels in their open and inactivated states [119]. Hesperidin also inhibits glutamate release and provides neuroprotection against kainic acid-induced excitotoxicity in the rats hippocampus by inhibiting Ca^2+^ channels [120]. In addition, it has been shown that hesperidin can improve ileus by regulating of Ca^2+^ channels [121]. Hesperidin treatment and TRPV1 channel inhibition offer a potential therapeutic opportunity for preventive and therapeutic strategies in the treatment of migraine [122]. The modulatory effects of hesperidin on TRP channels was shown in several studies [123,124].

#### 3.2.25. Icariin

Flavonoid glucosides, such as icariin, have been isolated from *Epimedium* species (*Berberidaceae*)*.* It has been demonstrated that icariin decreases intracellular calcium overload by inhibiting I_Na_ and I_CaL_ in the myocardium, exhibiting antiarrhythmic effects [78,125].

#### 3.2.26. Kaempferol

The flavonoid kaempferol is widely present in food plants such as cabbage, broccoli, kale, beans, tea and tomatoes [44]. Experimental studies have shown that kaempferol enhances endothelium dependent relaxation in the goat and porcine coronary artery, primarily through the activation of calcium-activated K^+^ channels [44,126,127]. It also exerts analgesic and anti-inflammatory effects *via* TRPV1 in a rat model. TRPV1 inhibition in the peripheral nerve endings reduce calcium and sodium influx, inhibiting nociceptive sensitization [128–131].

#### 3.2.27. Magnolol

Magnolol, a polyphenolic compound isolated from Houpu, the bark of *Magnolia officinalis*, has demonstrated its neuroprotective effects both *in vitro* and *in vivo* [132]. Magnolol acts as a dual inhibitor of voltage-gated sodium and potassium channels, which may partly explain its neuroprotective actions [132]. Magnolol also blocks Ca^2+^ channels, thereby inhibiting IP_3_ receptor type 1 and regulating IP_3_-mediated Ca^2+^ release from intracellular stores. Additionally, it activates calmodulin (CaM), leading to the suppression of SK channels. Furthermore, magnolol downregulates PKC, which promotes the opening of BKCaα1 and BKCaβ3 channels while closing BKCaβ4 channels. These combined mechanisms contribute to the modulation of intestinal ion secretion [133]. In rats, Zhang *et al*. [134] showed that magnolol inhibits colonic motility by down regulating voltage-gated LTCC in the colonic smooth muscle cells. It has been demonstrated that magnolol inhibits transient receptor potential cation channel C4 (TRPC4) to reduce calcium load and cause smooth muscle relaxation [135].

#### 3.2.28. Matrine

Matrine, a quinolizidine alkaloids widely distributed in *Sophora flavescens*, *Sophora alopecuroides*, and other legumes, exhibits antiarrhythmic effect [90]. It prolongs action potential duration and the effective refractory period, decreases heart rate, enhances myocardial contractility, and inhibits ectopic rhythms and atrioventricular reentry by blocking potassium, sodium, and calcium channels [136]. Studies have shown that matrine has concentration-dependent effects on hERG expression [137,138] in rat cardiomyocytes. At low concentrations (1 μmol/l), matrine promotes hERG expression, whereas at higher concentrations (100 μmol/l), it suppresses hERG expression, prolongs action potential duration and the effective refractory period of ventricular myocytes, slows spontaneous discharge frequency, and reduces the occurrence of ectopic rhythms [139]. In guinea pig ventricular myocytes, matrine (100 μmol/) shortens the ouabain-induced prolonged action potential and prevents the ouabain-induced increase in I_CaL_ and Ca^2+^ transients. This protective mechanism may involve matrine-mediated inhibition of I_CaL_, thereby reducing calcium overload by competing with ouabain for its binding sites [140]. It has been shown that chronic administration of matrine significantly improves left atrial conduction function, inhibits atrial myofibroblast differentiation, and reduces the duration of atrial fibrillation episodes in rat models. These effects appear to be associated with increased I_CaL_ density and Ca_v_1.2 protein expression in atrial myocyte membranes [141].

#### 3.2.29. Nantenine

Alkaloid nantenine is found in plant *Nandina domestica* and several *Corydalis* species. In animal studies, nantenine acts as an antagonist of the α-adrenergic receptors and the 5-hydroxytryptamine (serotonin) receptor 2A (5-HT2A) [142]. Nantenine has also been shown to exert antiepileptic effects by reducing Ca^2+^ influx into cells through inhibition of Ca^2+^ channels [143].

#### 3.2.30. Naringin

Grapefruit juice has a characteristic bitter taste, and its major flavonoid is the flavanone naringin; making it an important dietary source of flavonoids [144]. In mouse hearts, naringin has a negative inotropic effect, which may result from a reduction in both I_Na_ and I_CaL_ [144].

#### 3.2.31. Neferine

Neferine, an alkaloid extracted from the seeds of *Nymphaeaceae lotus* species, exhibits vasorelaxant, antihypertensive, and antiarrhythmic effects [145]. These properties result from its ability to reduce Na^+^ and Ca^2+^ influx while increasing the K^+^ efflux. Neferine has also been shown to prolong QT interval and effectively slow the heart rate [146].

#### 3.2.32. Osthole

The coumarin derivative osthole (7-methoxy-8-(3-methyl-2-butenyl) coumarin) is an important bioactive compound found in several medicinal plants and herbs used in traditional medicine. It is clinically utilized and exhibits a wide range of pharmacological and biochemical activities [71]. Osthole has been shown to alleviate epilepsy in mice by increasing K_v_1.2 expression in neurons of the hippocampus CA3 region [143]. It has been shown that osthole alleviates neuropathic pain by suppressing astrocytes activation and associated inflammatory responses *via* the PKCδ/TRPV4 signaling pathway [147]. One study reported that osthole is a potent non-electrophilic agonist of TRPA1 [148].

#### 3.2.33. Orientin

The flavonoid monomer orientin is found in several medicinal plants, including *Ocimum sanctum*, bamboo leaves, and *Calendula officinalis*. Orientin has demonstrated antioxidative, antiapoptotic, antithrombotic and antiarrhythmic effects in experimental models, but it is not an established clinical drug [149,150]. Orientin by blocking LTCC promote cardiac artery dilation [151]. Furthermore, orientin has been reported to inhibit multiple ionic currents in cardiomyocytes including I_Na_, I_to_, and I_CaL_ and to modify their kinetic properties [152].

#### 3.2.34. Oxymatrine

Oxymatrine is an alkaloid isolated from the traditional Chinese herb *Sophora flavescens* [153]. Its beneficial antiarrhythmic effect may be related to its ability to shorten action potential duration by reducing I_CaL_, increasing I_to_, and blocking I_K1_ [153]. In addition, the antinociceptive effects of oxymatrine mediated through inhibition of voltage-activated K^+^ channel [154].

#### 3.2.35. Puerarin

Puerarin is a flavonoid compound with antiarrhythmic effect [155], which is extracted from dried root of *Pueraria puerariae*. It has been shown to decrease heart rate, to suppress cardiomyocyte automaticity, and to prolong the effective refractory period and action potential duration [156]. Puerarin also blocks Na^+^ current, which contributes to its antiarrhythmic effect [157]. Puerarin acts as a TRPV4 agonist, induces endothelium-dependent vasodilation in mouse mesenteric arteries, and attenuates blood pressure in high-salt-induced hypertensive mice [158]. Puerarin ameliorates cisplatin-induced ototoxicity and blocks cellular apoptosis by inhibiting activated TRPV1/IP3R1/p65 pathway, blocking the induction of calcium overload and excessive ROS production [159]. On the other hand, some studies revealed the inhibitory effects of puerarin on TRP channels [160,161].

#### 3.2.36. Protopine

The isoquinoline alkaloid protopine is obtained from the *Corydalis tubers*. It has been reported to exhibit antiarrhythmic, antihypertensive, negative inotropic and other cardiovascular protective effects [162]. It has been demonstrated that protopine inhibits I_CaL_ in a concentration-dependent manner, with no apparent effect on transient calcium current (I_CaT_) in isolated guinea pig ventricular myocytes [163].

#### 3.2.37. Quercetin

Quercetin, a flavonoid commonly found in fruits, vegetables, and leaves [164], exhibits cardioprotective, antihypertensive and other biological activities [165,166]. In rats, quercetin acts as an LTCC inhibitor, producing cardioprotective effects by reducing Ca^2+^ influx and myocardial contractility [167]. Additionally, isoquercetin has been shown to exert antiepileptic effects by modulating the GABA_A channel and Cl^−^ current [143]. It has also been shown that quercetin increase colonic contraction by stimulating calcium channels [168].

#### 3.2.38. Quinidine

Quinidine, an alkaloid derived from the bark of the cinchona tree, has been used to treat both atrial and ventricular arrhythmias [10]. It has been reported to block I_Ks_, I_to_, and ultrarapid delayed-rectifier potassium (I_Kur_) currents, and these actions contribute to its antiarrhythmic effects [169,170].

#### 3.2.39. Resveratrol

Resveratrol, extracted from dry roots of *Polygonum cuspidatum Sieb et Zucc*. (Polygonaceae) has antiarrhythmic properties [171]. Resveratrol regulates potassium, sodium and calcium ion channels, K_Ach_, and hHCN4, which is highly expressed in sinoatrial node pacemaker cells, where it generates the funny current (I_f_), thereby slowing heart rate, prolonging the effective refractory period of cardiomyocytes, and preventing early and delayed after depolarization [172,173]. It has been shown that resveratrol modulates diabetes-induced neuropathic pain, apoptosis, and oxidative neurotoxicity in mice through TRPV4 channel inhibition [174].

#### 3.2.40. Tetrandrine

Tetrandrine is a dibenzyl isoquinoline alkaloid derived from the rhizomes of *Trichosanthes* Merr*.* Chun*.* and from roots of *Stephania discolor*, *Stephania tetrandra* and *Aristolochia heterophylla*. It is recognized as an antiarrhythmic agent due to its ability to inhibit calcium, potassium, and sodium channels [175]. Tetrandrine can decrease the heart rate, inhibit atrioventricular conduction, and prolong cardiomyocytes effective refractory period [176].

## 4. Discussion

Ion channels play essential roles in regulating electrical properties and synchronized contraction of the heart. They are also essential for the function of central and peripheral nervous systems and the transmission of electrical signals among neurons. The digestive system also relies heavily on ion channels to regulate gastrointestinal motility, secretion and nutrient absorption. Therefore, disturbances in the normal activity of these channels, whether genetic or acquired, can impair the function of multiple organ systems.

Medicinal plants, as natural healing resources, have been widely used throughout history to manage various disorders and are commonly used as alternative medicines today. This review summarizes available information regarding the effects of medicinal plants and/or their active constituents on ion channels in different physiological systems.

In the cardiovascular system, the activation of voltage-dependent sodium channel initiates action potentials by allowing Na^+^ influx into cardiac myocytes. Potassium channels are responsible for repolarization, while calcium ions are involved in pacemaker activity, action potential formation, heart rate regulation, and myocardial contraction. Several medicinal plants or their phytochemicals affect multiple cardiac ion channels or different subunits of the same channel. For example, matrine inhibits sodium channel conductance as well as I_CaL_ and I_K_ [141,177]. Barbaloin blocks I_Na_ and I_Ca_ [77]. Berberine inhibits I_Kr_, I_Ks_, I_K1_, hHCN4 currents and both L-type and T-type calcium channels [82,178,179], contributing to its antiarrhythmic effects. Several herbal compounds exert their cardioprotective or antiarrhythmic effects by modulating potassium channels, such as puerarin [157], acacetin [180], artemisinin [181], and protopine [163] through channel blockade or *Melissa officinalis* [25] and ginsenoside [112] through potassium channels activation. In addition, components such as 6-gingerol [55], icariin [125], and 8-gingerol [59] show antiarrhythmic effects, while baicalein [182], ellagic acid [91], and quercetin [183] protect against myocardial injury by blocking calcium channels. However, some agents, such as *Aralia elata* [14], increase calcium channel conductance and promote cardiomyopathy. The effects of plants or their effective compounds on cardiac ion channels were summarized in Table 2.

Based on the findings of various studies, it can be suggested that different herbal medicines or their derivatives exert their effects on neurological disorders through decreasing or increasing the conductance of specific ion channels (Table 3). For example, curcumin and naringenin exert anti-nociceptive and analgesic effects by inhibiting sodium channels [46,184]. Alpha-bisabolol show similar effects by blocking sodium and potassium channels [185], whereas lavender mediates its analgesic action through modulation of calcium channels and interaction with NMDA receptors [18]. The anxiolytic effects of estragol has been attributed to its sodium-channel blocking effects [186]. In addition, several herbal agents, such as asarone, nantenin and ginsenoside exhibit anti-epileptic properties through attenuating calcium channel conductance [73,187,188]. Components such as berberine [86], linalool [19], safranal [34], kaempferol [128,131], osthole [147] and resveratrol [174] exhibit analgesic effects, and magnolol [135] causes smooth muscle relaxation by inhibiting TRP channels. However, capsaicin [189], eugenol [105,106] and epigallocatechin-3-gallate [102,103] activate TRP channels and temporary enhance nociceptive signaling, vasodilation and blood pressure reduction.

Despite the extensive use of plant-derived compounds for treating gastrointestinal disorders in traditional medicine, knowledge of their mechanisms, particularly their effects on ion channels remains limited. However, it is reported that some herbal agents such as magnolol inhibit colonic motility by downregulation of LTCC [134]. Ginsenoside and eugenol exhibit antidiarrheal effects through potassium channels modulation [190] and calcium channels inhibition [191], respectively, in the gastrointestinal smooth muscles, thereby contributing to the regulation of gastric motility and alleviation of indigestion symptoms [190].

## Gaps in Research and Foresight

Despite the substantial amount of information available in this field, our understanding of how medicinal plants influence ion channels remains limited, much like seeing only the tip of a floating iceberg. Given the diversity of subunits within each ion channel type, the effects of plant-derived components on each specific subtype must be investigated in detail. Furthermore, considering the distribution and diversity of ion channels subunits, which vary among tissues, these compounds may elicit distinct outcomes depending on the tissue type involved. These require comprehensive, large-scale studies to determine how various phytochemicals modulate individual channel subtypes and how these interactions translate to physiological effects on organ function and excitable cells. In addition, the existing findings are derived from experimental studies and due to the lack of clinical validation, the capacity to use laboratory results in real-world clinical settings is limited. For example, many herbal polyphenols, such as flavonoids, are characterized by inherently low oral bioavailability, largely due to poor intestinal absorption, extensive first-pass metabolism, and rapid systemic clearance [192]. As a result, the plasma concentrations achievable in humans following dietary intake or oral supplementation are typically far lower than the concentrations commonly used in *in vitro* assays, which frequently range from ≥10–100 μM. This discrepancy is important to acknowledge when interpreting mechanistic findings, as some of the ion channel modulatory effects reported at high micromolar concentrations may not be replicable under physiological *in vivo* conditions. Nevertheless, such data remain valuable for identifying potential molecular targets and for understanding the pharmacodynamic potential of these compounds under conditions of enhanced delivery or structural optimization.

Therefore, further studies are essential to elucidate the molecular mechanisms and tissue-specific actions of medicinal plants. Consequently, comprehensive clinical and pharmacological studies must be conducted to confirm the bioavailability, efficacy, doses and safety of these compounds for clinical uses. Such studies will be invaluable for clarifying the broader implications of phytochemicals in complementary medicine and their clinical potential in treating a range of disorders, such as cardiovascular, neurological, and gastrointestinal diseases.

## Conclusion

Overall, this review highlights how specific phytochemicals in medicinal plants interact with a variety of ion channels. Certain plants or their active constituents can influence multiple channel types or subunits, whereas others act more selectively. Given the diversity of ion channel subunits, their tissue-specific distribution, and their distinct activation mechanisms under physiological conditions, it is clear that herbal medicines can produce a wide array of tissue-dependent physiological and pharmacological effects. Consequently, the use of herbal agents may lead to different outcomes depending on the target tissue and the specific channels involved.

## Figures and Tables

**Table 1:** Classification, synonyms, localization, and major characteristics of ion channels

Type of Channel	Synonyms/ Abbreviation	Main Ion Conducted	Localization	Key Characteristics / Function	Clinically Relevant Inhibitors / Modulators
**Voltage-gated sodium channels**	Na_V_ (e.g., Na_V_1.5 in the heart)	Na^+^	Plasmalemmal	Initiate and propagate action potentials (rapid depolarization)	Lidocaine, tetrodotoxin
**L-type voltage-gated calcium channels**	Ca_V_1.x (Ca_V_1.1 –Ca_V_1.4); LTCC (L = long, long-lasting, large)	Ca^2+^	Plasmalemmal (T-tubules, sarcolemma)	Long-lasting Ca^2+^ influx; excitation-contraction coupling	Nifedipine, verapamil, diltiazem
**T-type voltage-gated calcium channels**	Ca_V_3.x	Ca^2+^	Plasmalemmal	Low-voltage-activated; pacemaker activity	Mibefradil, ethosuximide
**P/Q-type voltage-gated calcium channels**	Ca_V_2.1	Ca^2+^	Presynaptic terminals	Neurotransmitter release	ω-Agatoxin IVA
**N-type voltage-gated calcium channels**	Ca_V_2.2	Ca^2+^	Neurons	Neurotransmitter release	ω-Conotoxin GVIA
**R-type voltage-gated calcium channels**	Ca_V_2.3	Ca^2+^	Neurons, endocrine cells	Synaptic transmission, hormone release	SNX-482
**Voltage-gated potassium channels**	K_V_	K^+^	Plasmalemmal	Repolarization, control of excitability	4-aminopyridine tetraethylammonium (TEA)
**Inwardly rectifying potassium channels**	K_ir_	K^+^	Plasmalemmal	Maintain resting potential	Barium ions (Ba^2+^)
**ATP-sensitive potassium channels**	K_ATP_	K^+^	Plasmalemmal (heart, pancreas)	Couple metabolism to excitability	Glibenclamide, diazoxide
**Acetylcholine-activated potassium channels**	K_ACh_	K^+^	Plasmalemmal	Parasympathetic regulation of heart rate	Muscarinic antagonists
**Small-conductance Ca** **^2+^** **-activated K** **^+^** ** channels**	SK	K^+^	Plasmalemmal	Ca^2+^-dependent hyperpolarization	Apamin
**Big-conductance Ca** **^2+^** **-activated K** **^+^** ** channels**	BK (Maxi-K, KCa1.1)	K^+^	Plasmalemmal, smooth muscle	Link Ca^2+^ to membrane potential; regulate vascular tone	Paxilline, iberiotoxin
**Two-pore domain K** **^+^** ** channels**	K2P (TREK, TASK)	K^+^	Plasmalemmal	Background (leak) K^+^ current; set resting potential	Volatile anesthetics, bupivacaine
**Chloride channels**	ClC, CFTR, CaCC	Cl^−^	Plasmalemmal	Volume regulation, secretion, excitability	Niflumic acid, CFTR modulators
**Ca** **^2+^** **-activated chloride channels**	TMEM16A (ANO1)	Cl^−^	Plasmalemmal	Smooth muscle contraction, epithelial secretion	Niflumic acid
**Ligand-gated chloride channels**	GABA_A, glycine receptors	Cl^−^	Plasmalemmal (neurons)	Inhibitory neurotransmission	benzodiazepines, picrotoxin
**Proton channels**	HVCN1	H^+^	Plasmalemmal	Regulate pH, NADPH oxidase activity	Zinc ions (Zn^2+^)
**Transient receptor potential channels**	TRP (TRPC, TRPV, TRPM, etc.)	Ca^2+^, Na^+^	Plasmalemmal	Sensory transduction, temperature, osmotic stress	2-APB, ruthenium red
**Mechanosensitive / stretch-activated channels**	Piezo1, Piezo2, TREK-1	Cations (Na^+^, Ca^2+^, K^+^)	Plasmalemmal	Mechano-transduction, pressure sensing	GsMTx4 (spider toxin)
**Hyperpolarization-activated cyclic nucleotide-gated channels**	HCN	Na^+^, K^+^	Plasmalemmal (SA node, neurons)	Pacemaker (If) currents	Ivabradine
**Pannexin and connexin channels**	Pannexin, Connexin (gap junctions)	Ions, ATP	Plasmalemmal	Intercellular communication, paracrine signaling	Carbenoxolone
**Ryanodine receptors**	RyR1–RyR3	Ca^2+^	Intracellular (sarcoplasmic/ endoplasmic reticulum)	Release of Ca^2+^ from SR/ER stores	Ryanodine, caffeine
**Inositol 1,4,5-trisphosphate receptors**	IP_3_R	Ca^2+^	Intracellular (endoplasmic reticulum)	IP_3_-mediated Ca^2+^ release	Xestospongin C, 2-APB
**Store-operated calcium channels**	Orai1/STIM1 complex (SOCE)	Ca^2+^	Plasmalemmal-ER junction	Refill intracellular Ca^2+^ stores after depletion	SKF-96365, YM-58483
**Mitochondrial calcium uniporter**	MCU	Ca^2+^	Intracellular (mitochondrial membrane)	Controls mitochondrial Ca^2+^ uptake	Ru360

**Table 2:** The properties of compounds and plants.

Name	Type of compound	Compound chemical structure or plant picture	Source of compound	Chemical constituents of plants
**Salvia miltiorrhiza**	-	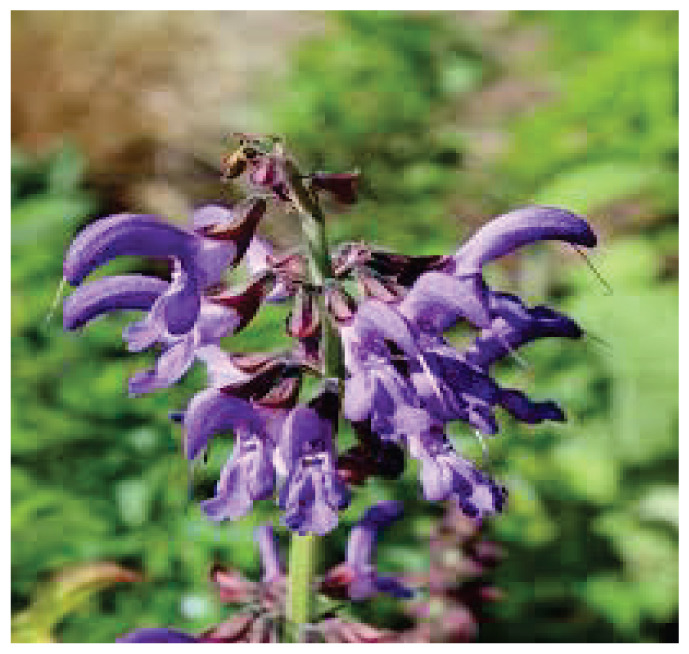	-	Salvianolic acid Dihydrotanshinone Miltirone Tanshinone I
**Lavender**	-	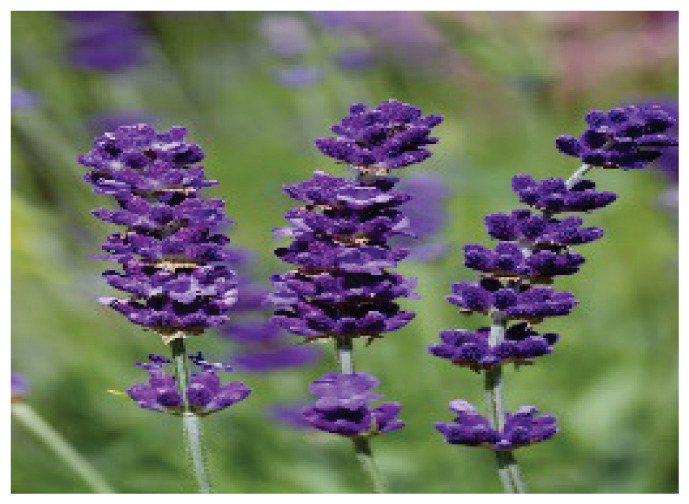	-	Linalyl acetate Linalool Tannins Caryophyllene
**Melissa officinalis (Lemon Balm)**	-	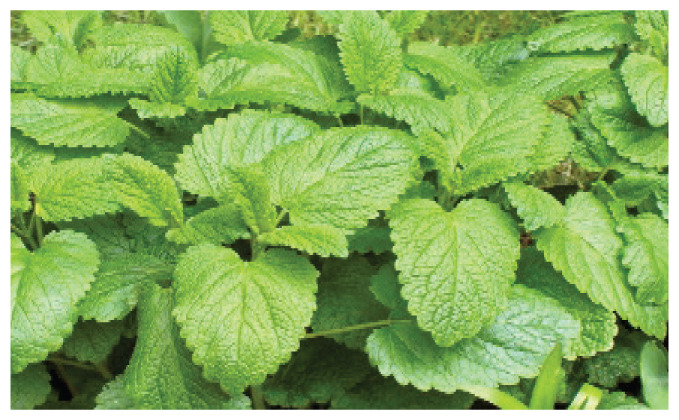	-	Eugenol Tannins Terpenes
**Aralia elata**	-	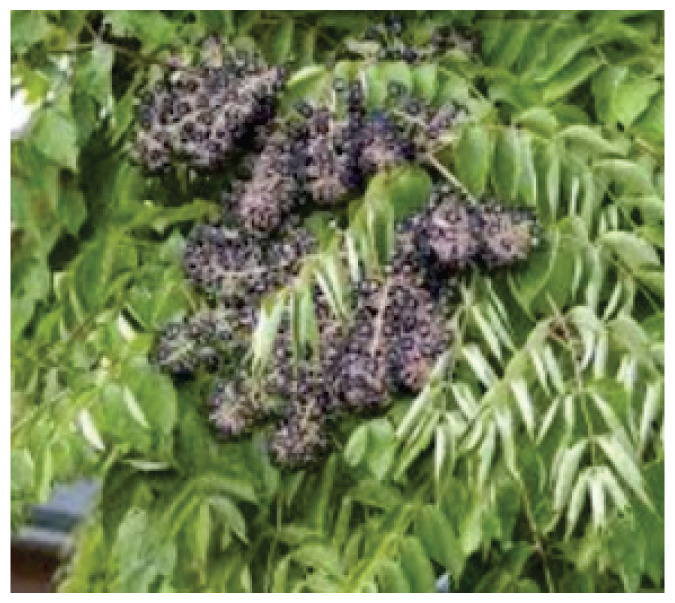	-	SilphiosideA Chikusetusaponin Ib Araloside A
**Saffron**	-	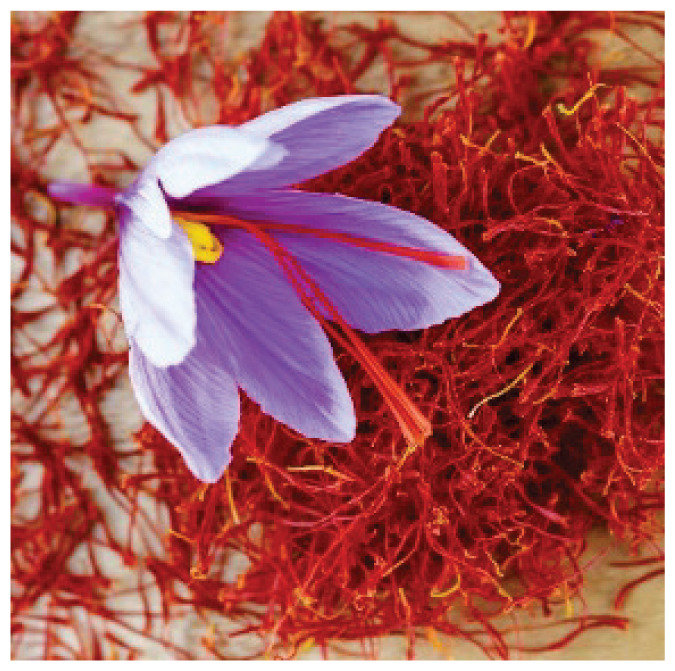	-	Picrocrocin Safranal Carotenoid Crocin
**Berberine**	Alkaloid	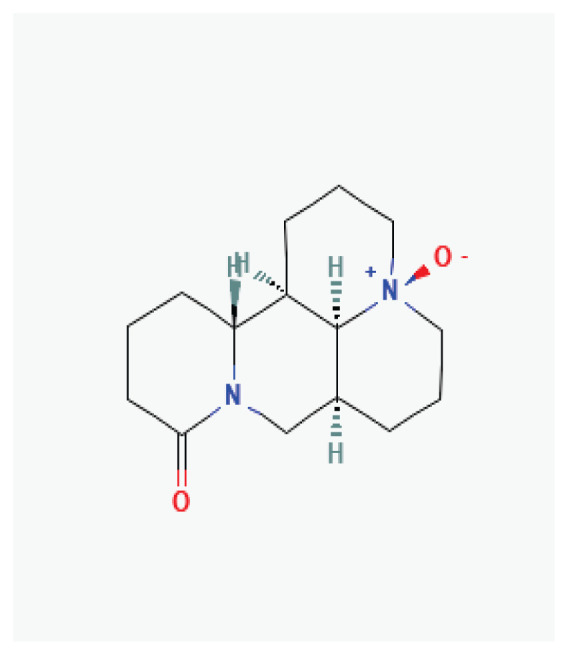	*Berberis vulgaris* *Berberis aristata* *Mahonia aquifolium* *Hydrastis canadensis*	-
**Martine**	Alkaloid	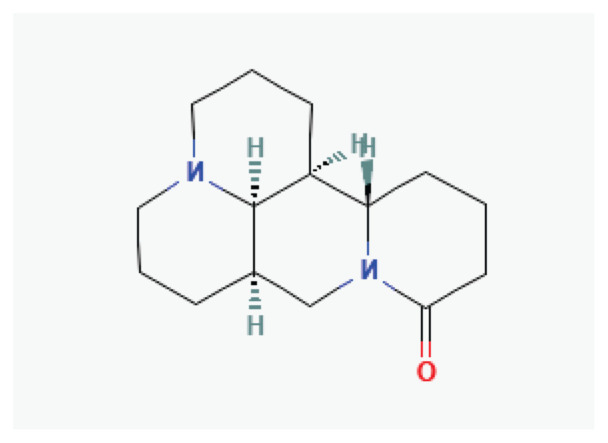	*Sophora flavescens*	-
**Guanfu base A**	Alkaloid	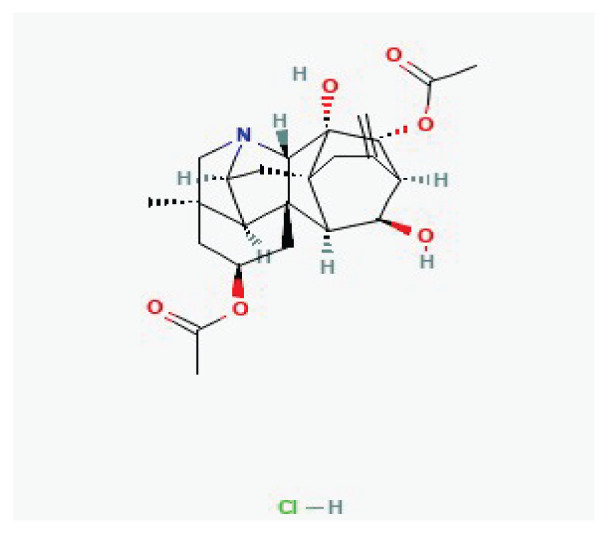	*Aconitum coreanum*	-
**Neferine**	Alkaloid	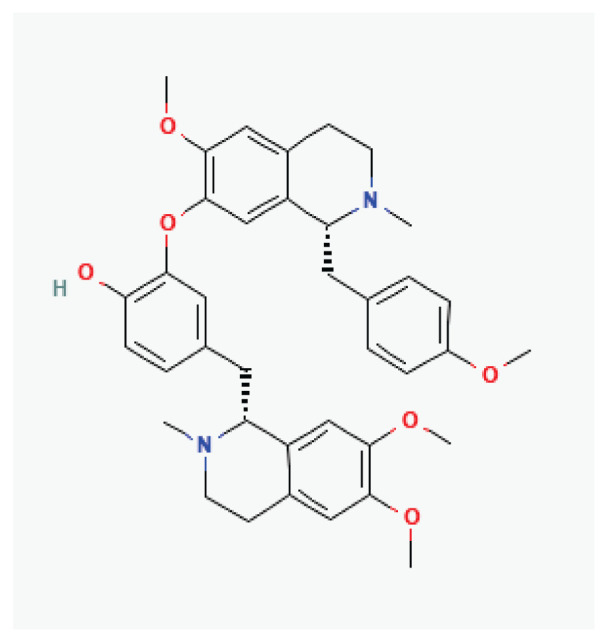	*Nelumbo nucifera*	-
**Aloperine**	Alkaloid	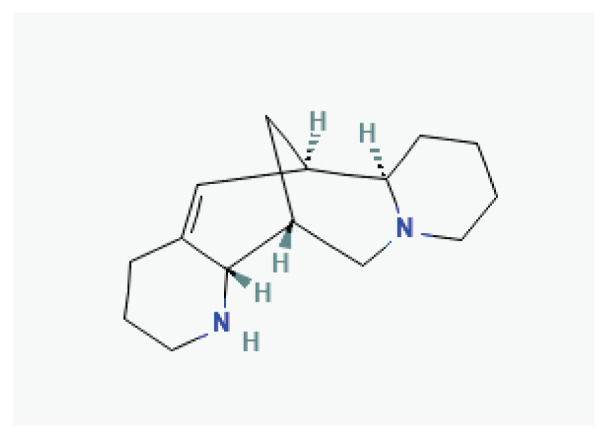	*Sophora alopecuroides*	-
**Protopine**	Alkaloid	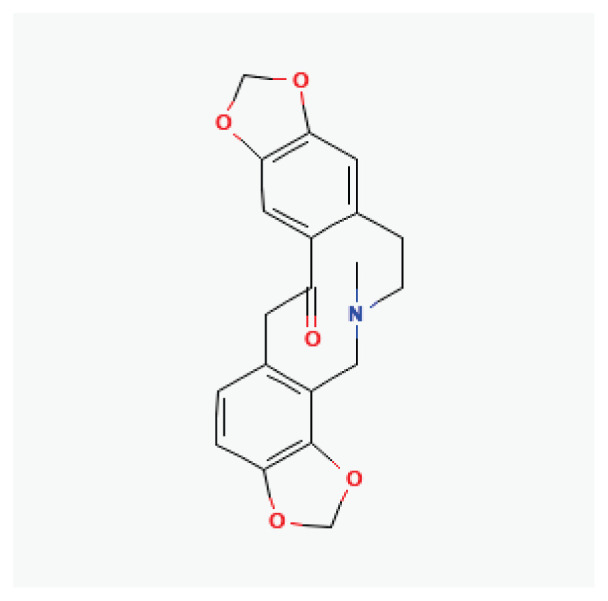	*Opium poppy* *Corydalis tubers*	-
**Tetrandrine**	Alkaloid	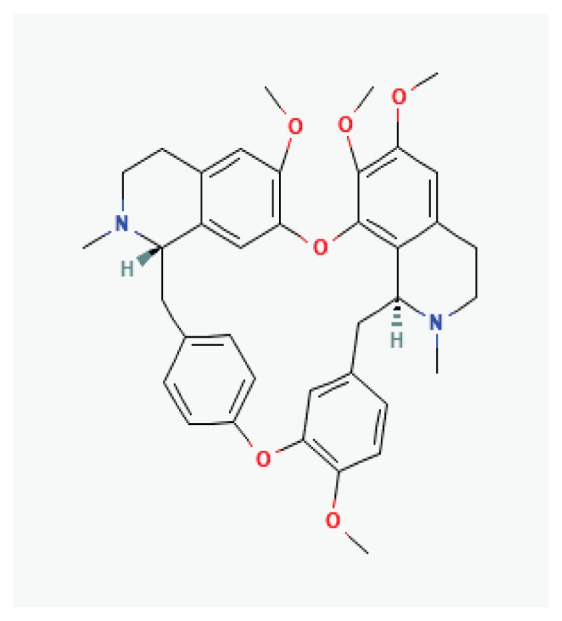	*Stephania tetrandra*	-
**Oxymatrine**	Alkaloid	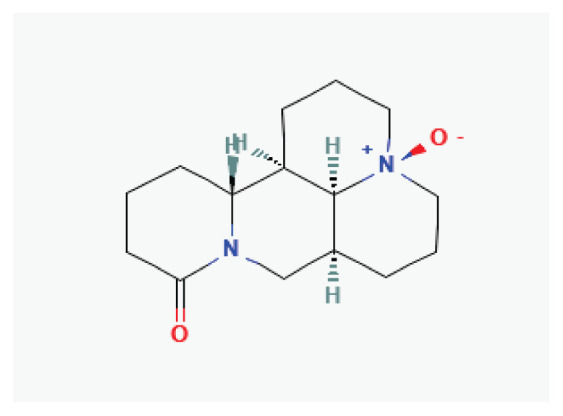	*Sophora flavescens*	-
**Dauricine**	Alkaloid	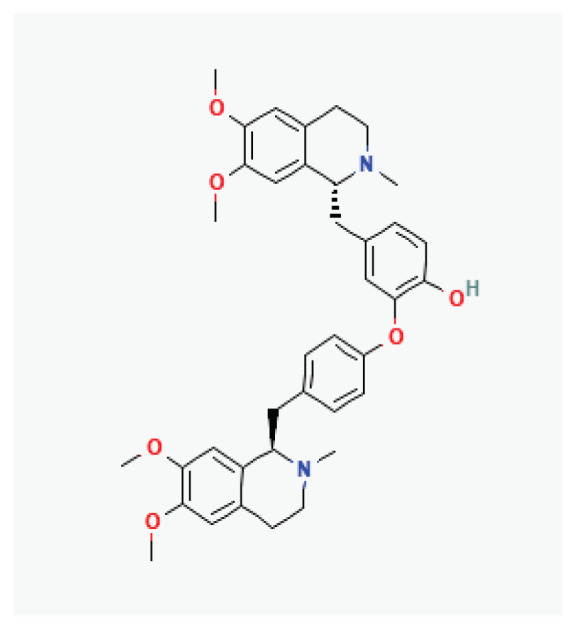	*Menispermum dauricum* *Menispermum canadense*	-
**Emodin**	Anthraquinone aglycone	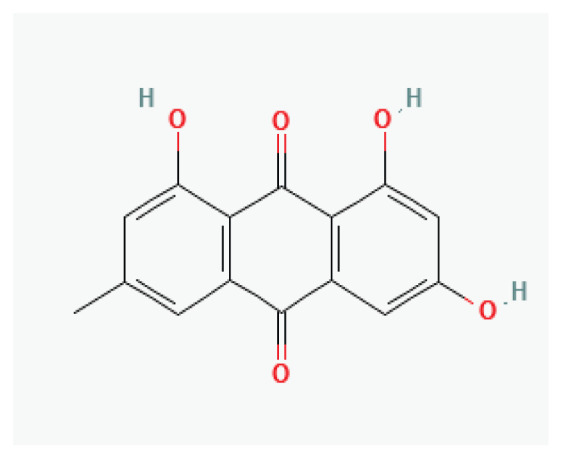	*Rhubarb* *Buckthorn*	
**Barbaloin**	Anthraquinone glycosyl	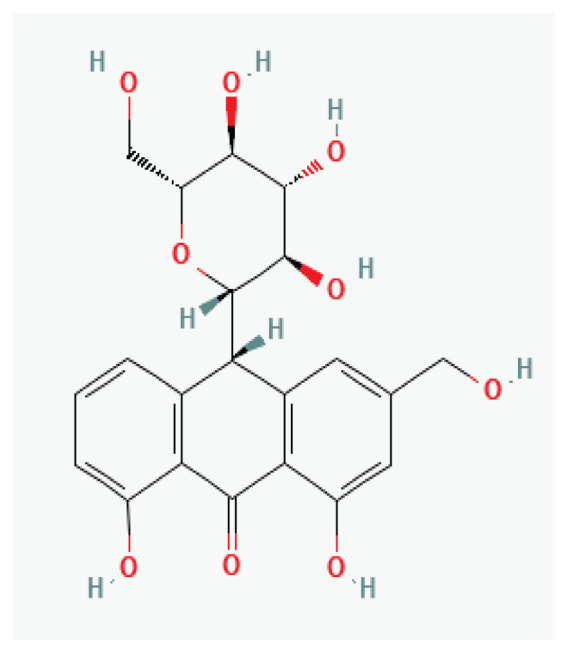	*Aloe species*	-
**Osthol**	Coumarin	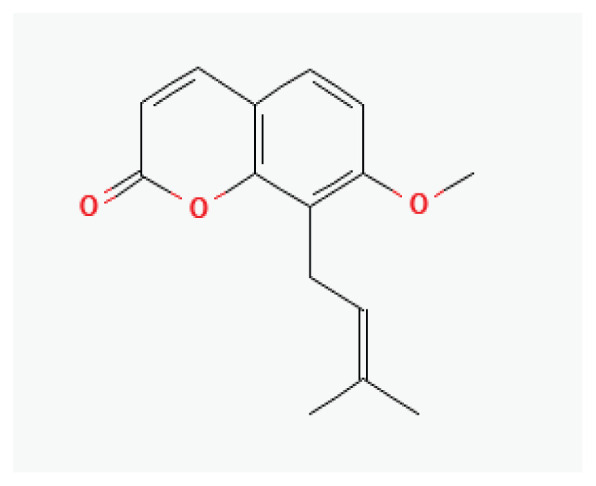	*Cnidium monnieri* *Angelica archangelica* *Angelica pubescens*	
**Epigallocatechin-3-Gallate**	Flavanol	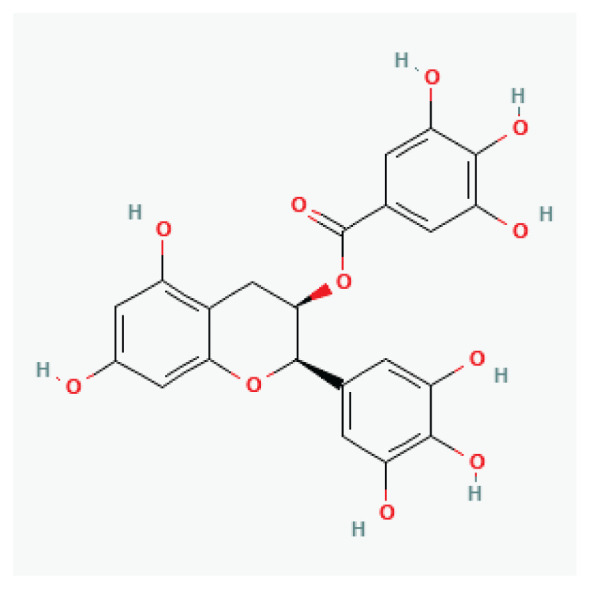	*Apple skin* *Plums* *Onions* *Hazelnuts* *Pecans* *Tea*	
**Naringenin**	Flavanone	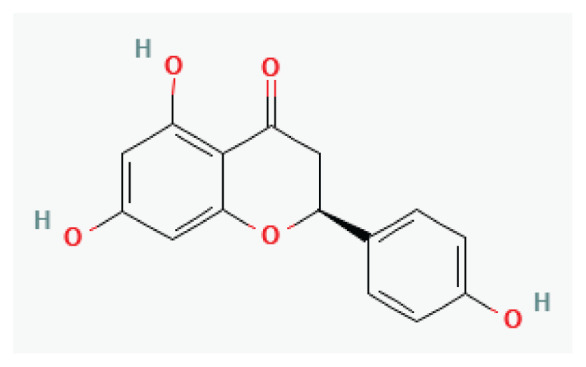	*Citrus fruits such as Grapefruit*	-
**Hesperidin**	Flavanone glycoside	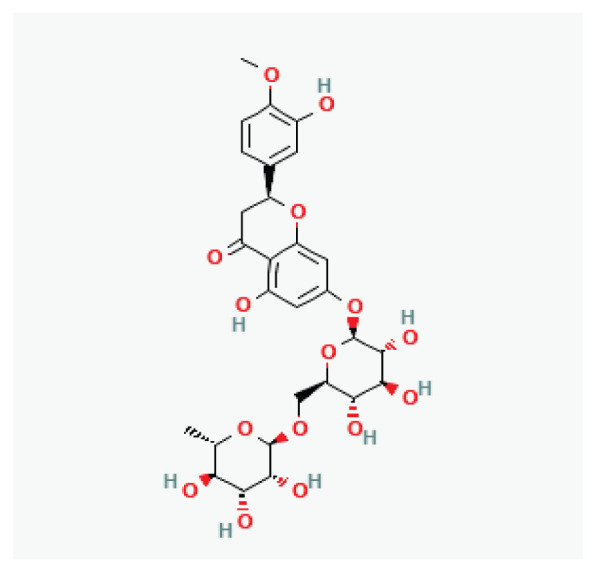	*Peel of citrus fruits*	-
**Acacetin**	Flavone	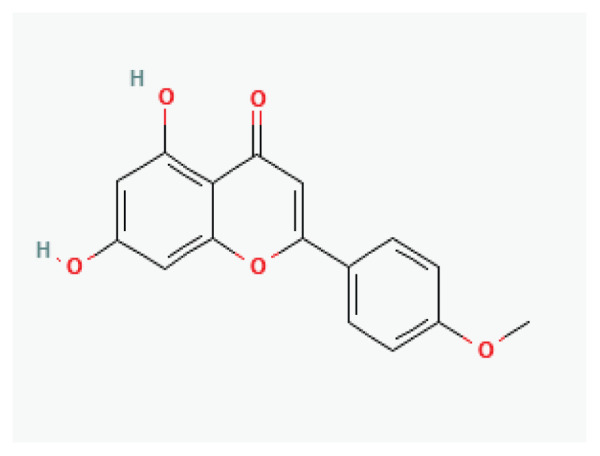	*Robinia* *pseudoacacia* *Turnera diffusa* *Betula pendula*	-
**Orientin**	Flavone	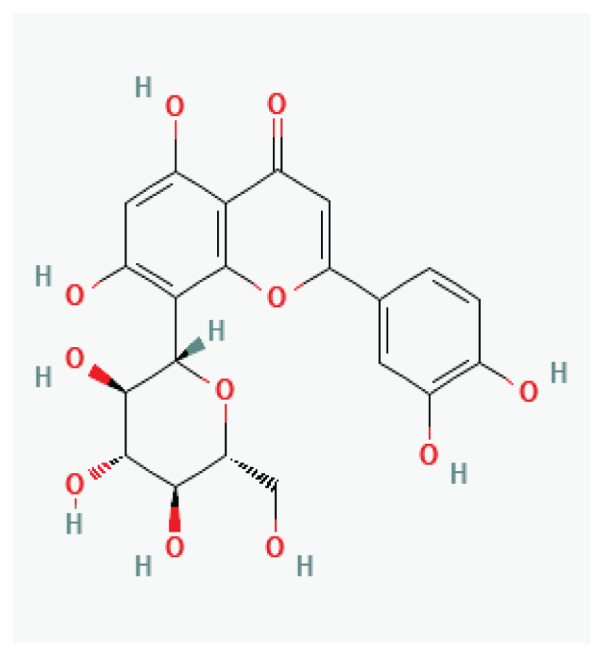	*Adonis vernalis* *Anadenanthera colubrina* *Anadenanthera peregrina* *Phyllostachys nigra*	
**Baicalein**	Flavone	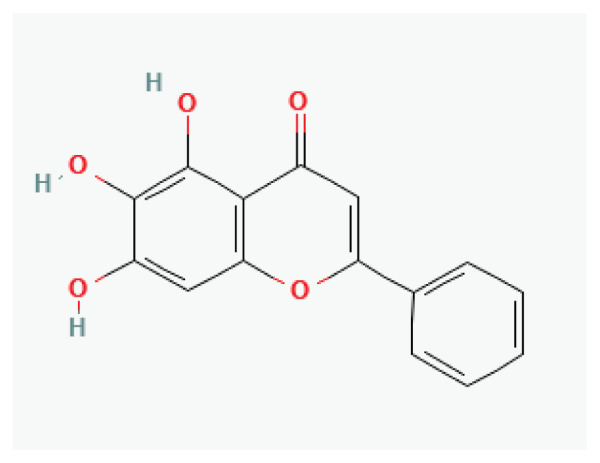	*Scutellaria baicalensis* *Scutellaria lateriflora*	-
**Quercetin**	Flavonol	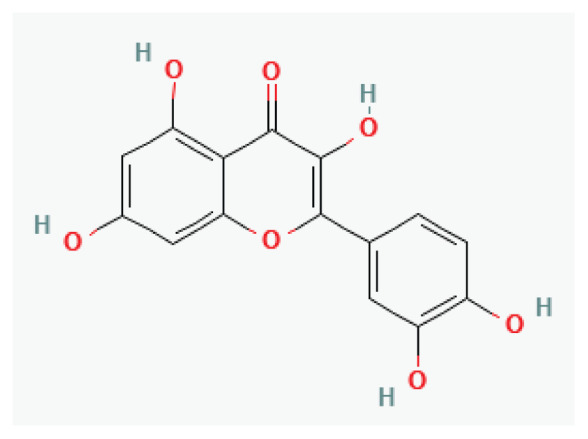	Many fruits, vegetables	-
**Kaempferol**	Flavonol	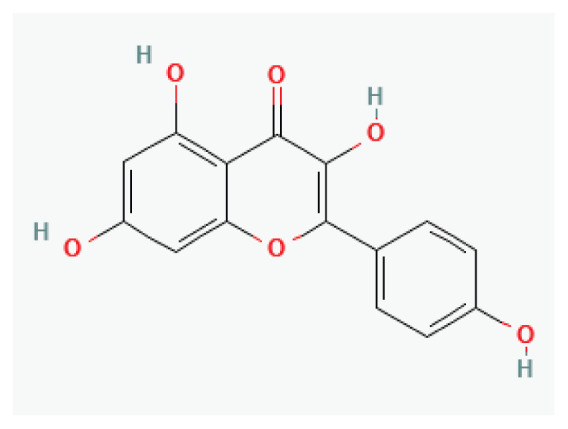	*Kale* *Beans* *Tea* *Spinach* *Broccoli*	
**Genistein**	Isoflavonoid	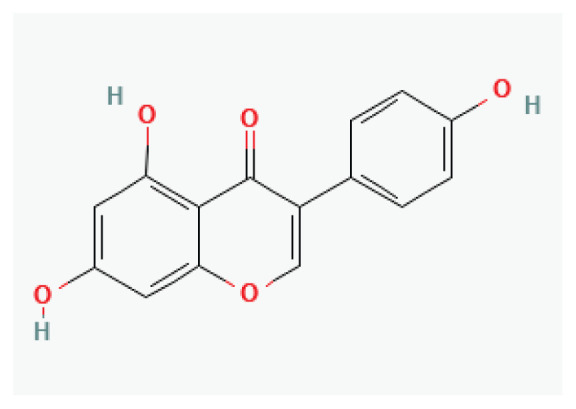	*Soybeans*	
**Puerarin**	Isoflavonoid	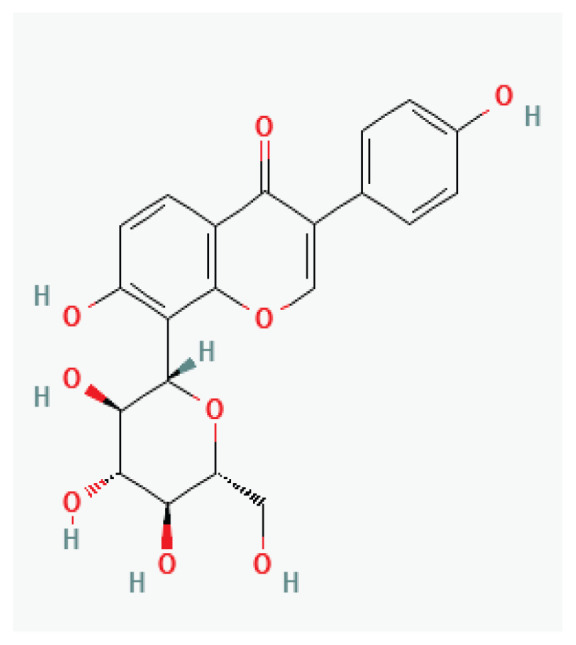	*Pueraria lobata* *Pueraria phaseoloides*	-
**Magnolol**	Lignan	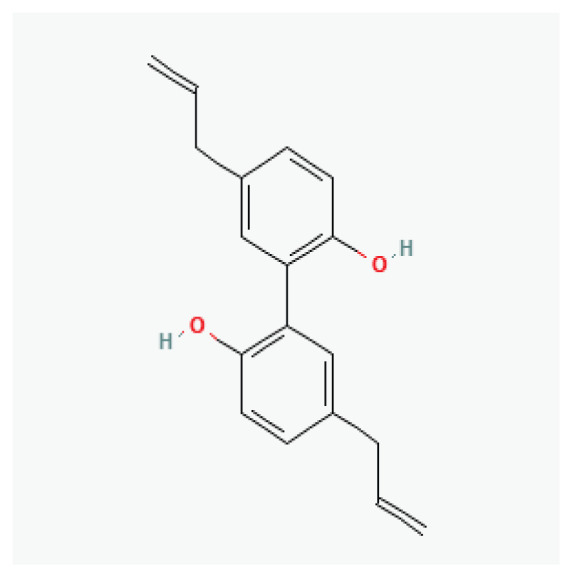	*Magnolia officinalis* *Magnolia grandiflora*	-
**Allicin**	Organosulfur compound	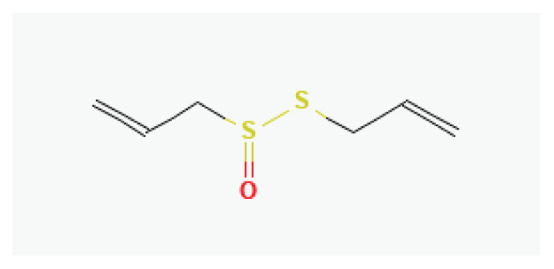	*Garlic* *Leeks*	-
**6-gingerol**	Phenolic phytochemical	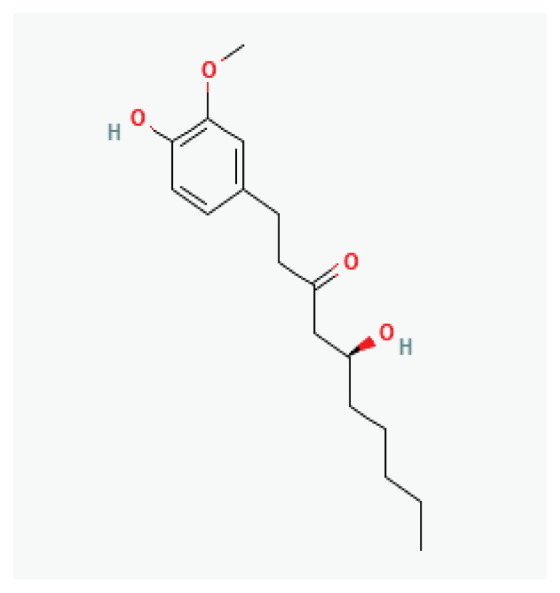	*Ginger*	-
**Eugenol**	Phenylpropanoid	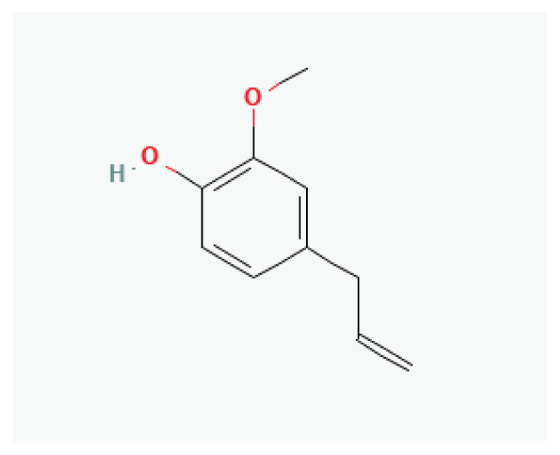	*Nutmeg* *Cinnamon* *basil* *bay leaf*	-
**Carvacrol**	Phenylpropanoid	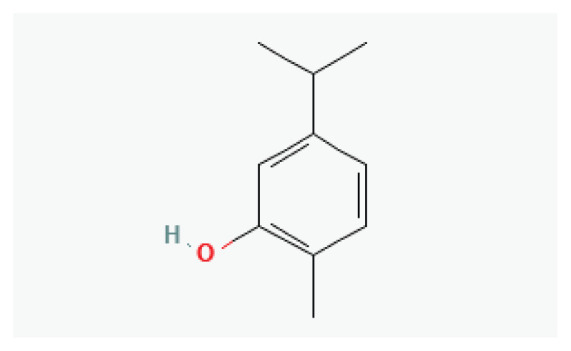	*Origanum vulgare* *Thyme* *Pepperwort* *Wild bergamot*	-
**Asarone**	Phenylpropanoid	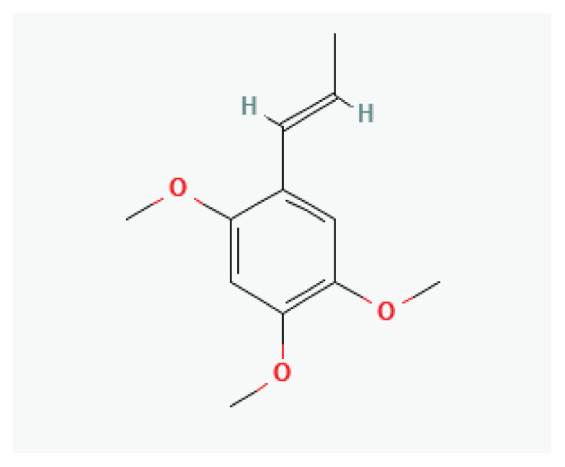	*Acorus* *Asarum*	-
**Estragole**	Phenylpropene	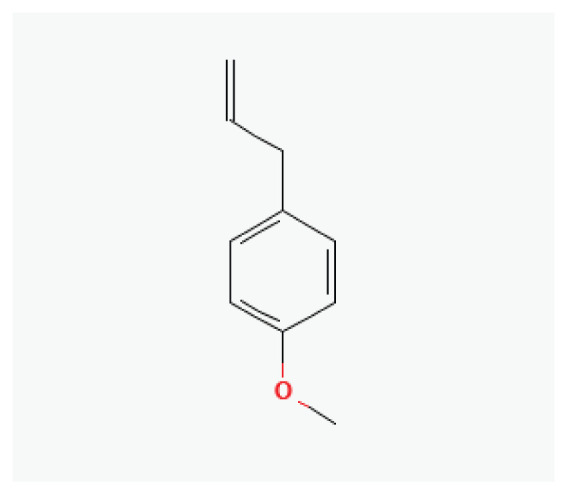	*Anise* *Fennel* *Bay* *Tarragon* *Basil*	-
**Curcumin (Turmeric)**	Polyphenol	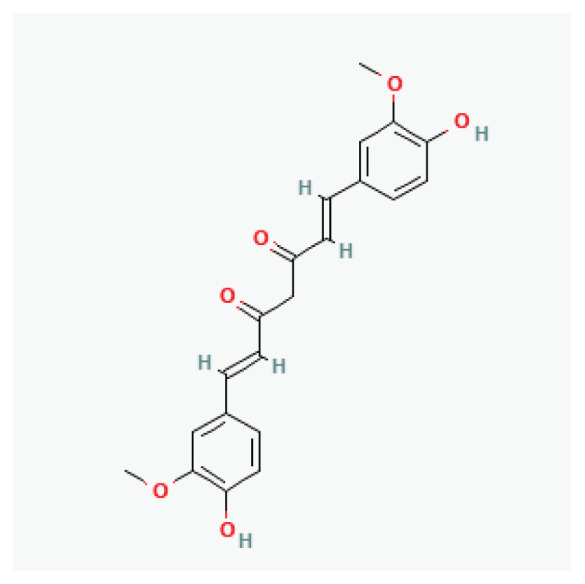	*Curcuma longa* *Ginger*	-
**Ellagic acid**	Polyphenol	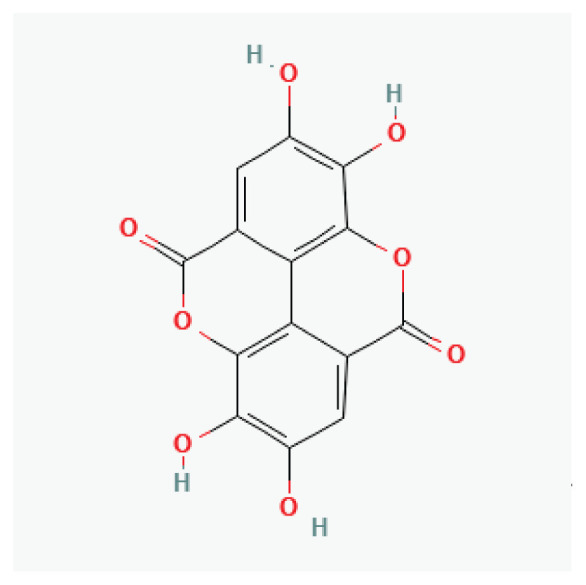	Numerous fruits and vegetables	-
**Resveratrol**	Polyphenol	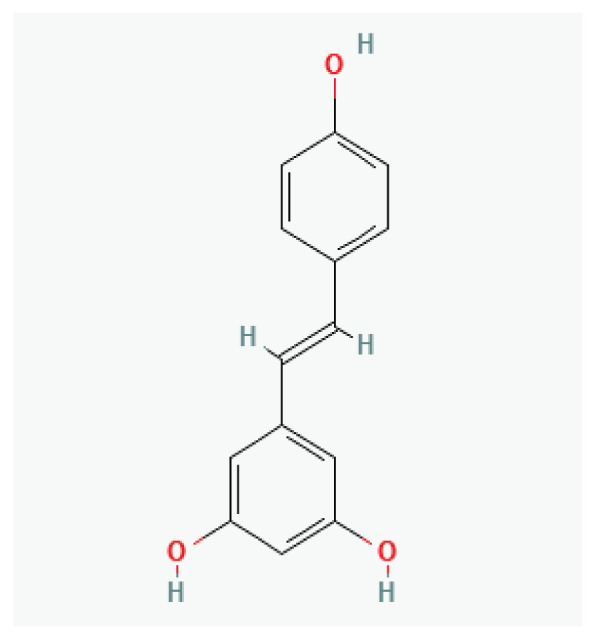	*Grapes* *Blueberries* *Raspberries* *Mulberries* *Peanuts*	-
**Salvianic acid A**	Polyphenolic acid	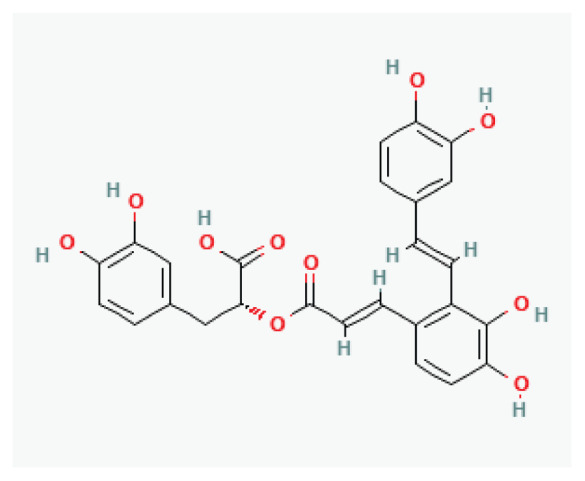	*Salvia miltiorrhiza*	
**Icariin**	Prenylated flavonol glycoside	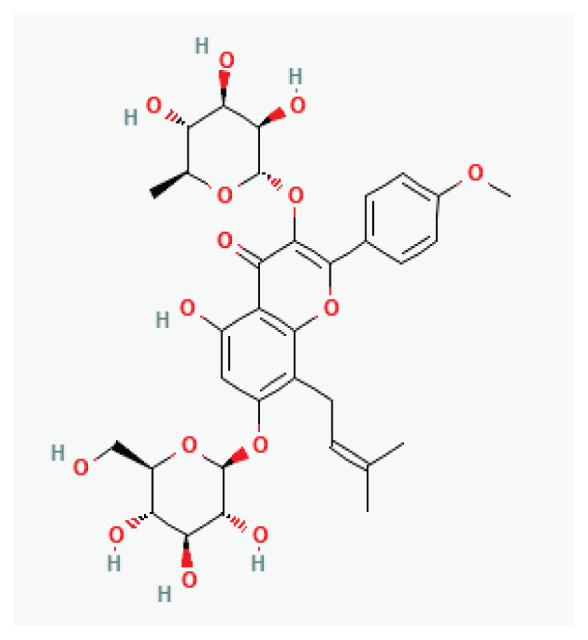	*Epimedium*	-
**α-(−)-Bisabolol**	Sesquiterpene alcohol	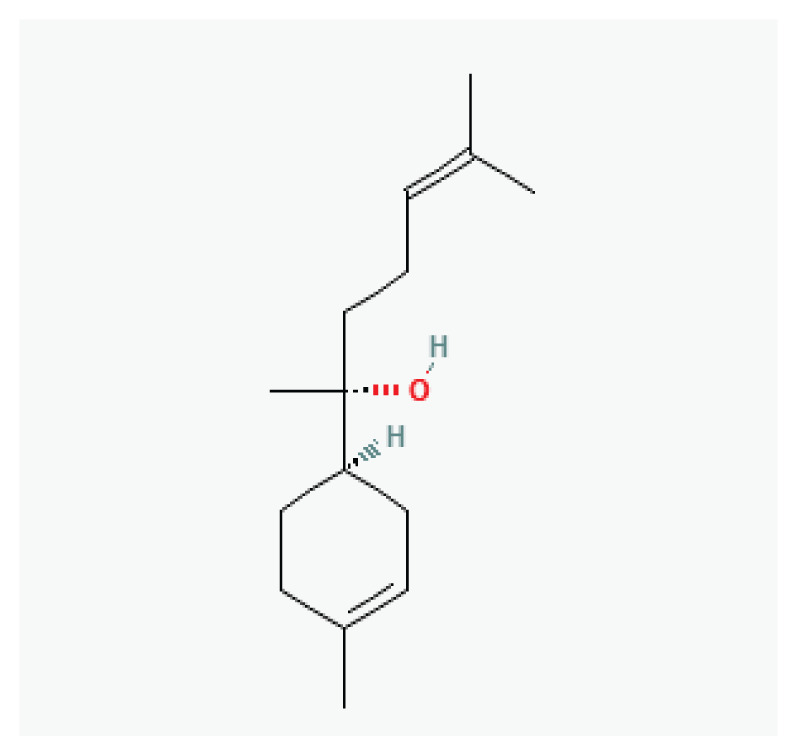	*Matricaria recutita* *Myoporum crassifolium*	-
**Artemisinin**	Sesquiterpene lactone	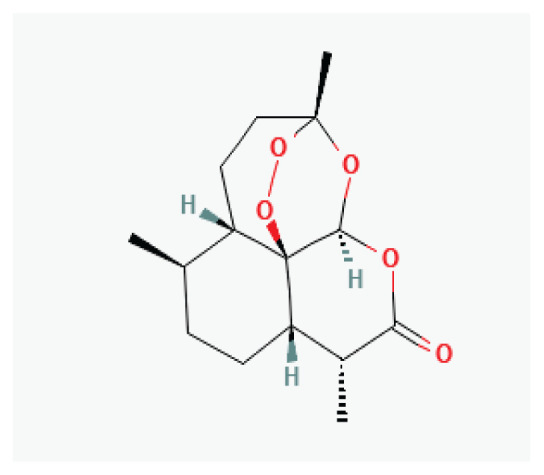	*Artemisia annua*	-
**Ginsenoside**	Steroid glycosides and triterpene saponins	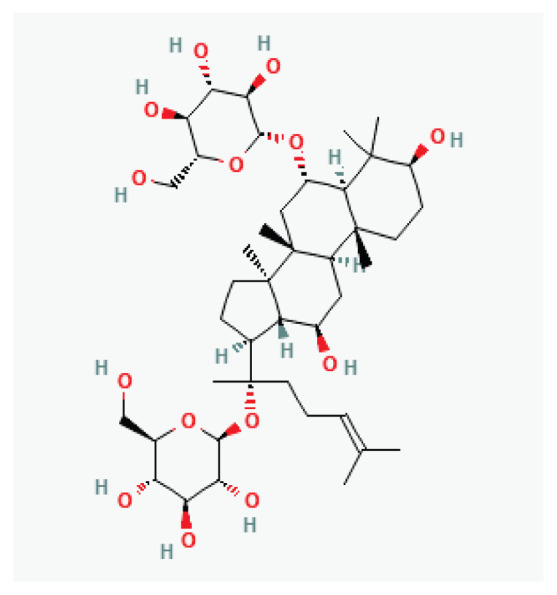	*Panax (ginseng)*	-
**Astragaloside IV**	Triterpenoid saponin	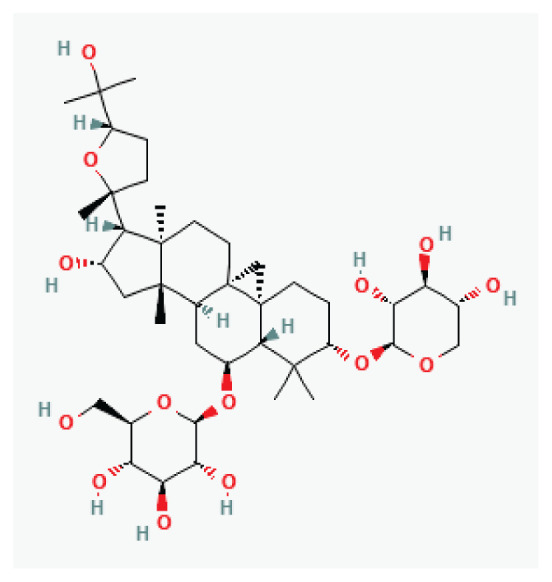	*Astragalus hoantchy* *Astragalus lepsensis*	-
**Glycyrrhizic acid**	Triterpenoid saponin	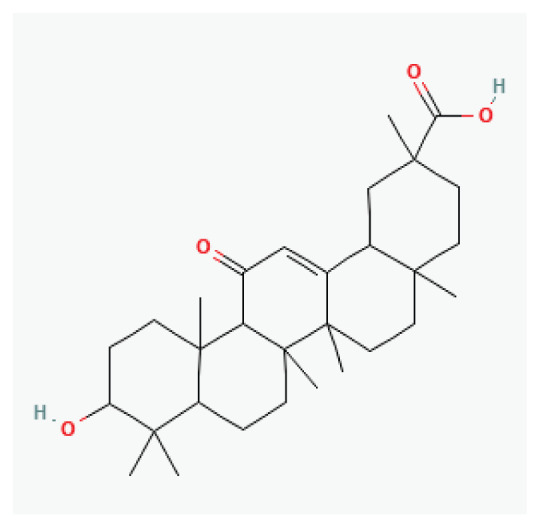	*Glycyrrhiza glabra*	-

**Table 3:** The effects of herbal compounds on cardiac ion channels

Compound	Effect on ion channel conductivity	Main effect	Mechanisms of action	Species	Dose/Concentration	Ref
**6-gingerol**	ICaL ↓	Antiarrhythmic effect	Increased action potential duration	Rat	300 μmol/l	[55]
**8-gingerol**	LTCC ↓	Antiarrhythmic effect	Myocardial anti-ischemic effects	Rat	10 and 20 mg/kg	[59]
**Acacetin**	IKur, IKr, KACh, Ito and SK↓ Ito ↓	Antiarrhythmic effect Improved atrial fibrillation	Increased action potential duration Increased refractory period of the fibrillating atrium	Dog Human Rabbit	5–10 μM 2.5, 5 and 10 mg/kg	[60, 180, 193]
**Aloperine**	IKr → INa ↓	Antiarrhythmic effect	Increased action potential duration	Rat	10 mg/kg 3, 10 and 30 μM	[68, 70]
**Aralia elata**	ICaL → Ito ↔	Improvement of cardiomyopathy	Increased left ventricular systolic pressure Increased contractility	Rat Dog	19.6 mg/kg 30 and 60 mg/kg	[14, 194]
**Artemisinin**	IK1 and Ito ↓ HCN ↓	Antiarrhythmic effect	Increased action potential duration	Dog Frog	5 and 50 μmol/l	[181, 195, 196]
**Astragaloside IV**	IK1 and IKATP → IK ↓ ICaL ↓ CaSR ↓	Antiarrhythmic effect Decreased infarct size	Increased action potential duration Reduced apoptosis	Guinea pig Rat	1×10-6 M 16, 32 and 64 μM	[74, 197–200]
**Baicalein**	ICaL ↓ BK →	Protection of against myocardial infarction injury	Dampening of intracellular calcium Reduced amplitude of voltage-dependent calcium channels currents	Mouse	30 and 60 mg/kg 100 μM	[75, 182]
**Barbaloin**	ICaL ↓ INa ↓	Antiarrhythmic effect	Reduced delayed and early after-depolarization	Rabbit	100 and 200 μmol/l	[77]
**Berberine**	IKr, IKs, IKATP and IK1 ↓ ICaL and ICaT ↓ hHCN4 ↓	Antiarrhythmic effect	Increased action potential duration Decreased rate of pacemaker and diastolic depolarization	Rat Guinea pig	3–100 μmol/l 10 and 20 mg/kg i.p. 10 and 30 μmol/l	[82, 178, 179, 201–203]
**Carvacrol**	TRPM7 ↓ IKv →	Antiarrhythmic effect	Decrease in conduction velocity	Rabbit Human	100 μM	[87, 204]
**Curcumin (Turmeric)**	INa, ICaL and IKr ↓	Prevention of ischemia/reperfusion-induced arrhythmias	Shortened action potential duration Suppressed early and delayed afterdepolarization	Rabbit	30 μmol/l	[42]
**Dauricine**	IKr and IKs ↓ ICaL ↓	Reduced calcium concentration and Inhibition of Ca^2+^-ATPase activity	Increased action potential duration Increased atrial effective refractory period	Guinea pig Rabbit	1, 3, 10, 30, 100 μmol/l	[90, 205, 206]
**Elatoside C**	SERCA2 → mPTPs ↓	Increases in left ventricular systolic pressure, ± dP/dtmax, and heart rate	Attenuating calcium overload	Rat		[93]
**Ellagic acid**	ICaL ↓	Protection against coronary artery diseases	Negative inotropic effect	Rat	23 nM	[91]
**Emodin**	BK and IK1 →	Improved hypertension	Aorta relaxation Reduced QT interval	Rat		[95, 207]
**Epigallocatechin-3-gallate**	ICaL ↓ IKr, IKs, Ito and ‘IKATP ↓ TRPA1 →	Antiarrhythmic effect	Reduced action potential duration	Rat	10 μM 30 μM	[100, 101]
**Genistein**	BK and IKur ↓ IKATP →	Improved atherosclerosis and hypertension	Vascular relaxation	Rat Human	50 μmol/l 30 μmol/l 5 mg/kg	[107, 208–210]
**Ginsenoside**	LTCC ↔ IKs → INa ↓ IK1 and IKv ↓	Antiarrhythmic effect	Reduced action potential duration Reduced amplitude	Guinea pig Rabbit	5, 10 and 20 mM	[111, 112]
**Glycyrrhizic acid**	INa ↓ ICaL ↓ Ito, IKr and IKs ↓	Antiarrhythmic effect	Reduced conduction speed Increased action potential duration Increased effective refractory period	Rat Guinea pig Ventricular myocytes Xenopus oocytes	10 mg/kg 3.5 mg/kg	[114, 211]
**Guanfu base A**	IKs and IKr ↓ Kir and Ito ↔ LTCC ↓	Antiarrhythmic effect	Reduced action potential amplitude Increased action potential duration	Human Dog Monkey, Rabbit	100, 400, 1000, 2500 μmol/l 25, 125, 250, 1000 μmol/l	[118, 196, 212, 213]
**Hesperetin**	INa ↓ IKs ↓	Antiarrhythmic effect	Decrease in conduction velocity	Human Rabbit Dog		[119, 214]
**Icariin**	LTCC ↓	Antiarrhythmic effect	Reduced delayed after depolarization	Rabbit	5 and 10 μM	[125]
**Kaempferol**	BK → IKv ↓	Attenuated hypertension	Vascular relaxation	Rat Goat Pig	10-7-10-4 M3 3 × 10-6 M	[44, 215]
**Matrine**	IKr ↕ IKM3 ↓ KCNB1 and KCNJ2 → LTCC→ INa ↓	Antiarrhythmic effect Reduced atrial fibrillation	Increased action potential duration Reduced action potential amplitude	Rat Guinea pig	1 and 100 μmol/l 15, 30, 45 mg/kg i.v. 50, 100, 200 mg/kg p.o.	[139, 177]
**Melissa officinalis**	LTCC ↓ IK→	Antiarrhythmic effect	Modulation of ECG and heart electrical system	Rat	50, 100 and 200mg/kg	[21, 25]
**Naringin**	ICaL ↓ INa ↓	Negative inotropic effects	Regulation of heart contractility	Mouse Rat	30–100 μM 10–30 μM	[127, 144]
**Neferine**	IKr ↓ ICaL ↓ INa ↓	Antiarrhythmic effect	Increased action potential duration Increased effective refractory period Reduced action potential amplitude	Rat In vitro	10 and 30 μmol/l	[216, 217]
**Orientin**	ICaL ↓ INa ↓ Ito ↓	Antiarrhythmic effect	Reduced action potential duration	Mouse	40 mg/kg	[152]
**Panax notoginseng saponins**	KCNN3 ↓ SK ↓	Antiarrhythmic effect Improved atrial fibrillation	Increased action potential duration Reduced calcium release from sarcoplasmic reticulum	Rat	150 mg/kg i.p.	[218, 219]
**Protopine**	ICaL ↓ ICaT ↔ IK1 and IK ↓ INa ↓	Antiarrhythmic effect	Increased action potential duration	Guinea pig	25, 50 and 100 μM	[163]
**Puerarin**	IKs, IK1 and Kir ↓ LTCC ↓ INa ↓	Antiarrhythmic effect Improved atrial fibrillation	Increased action potential duration	Rat Mice	0.01, 0.1 and 1.0 mmol/l 1.2 mmol/l	[196, 220–222]
**Quercetin**	ICaL ↓ KCNQ →	Anti-ischemic effects	Regulation of calcium homeostasis	Rat	60 mg/kg	[167, 183]
**Quinidine**	INa ↓ IKATP and IKr ↓	Antiarrhythmic effect	Increased action potential duration	Hamster Rat Guinea pig	9.7 μM	[223, 224]
**Resveratrol**	IKr and IKACh ↓ IKATP → ICaL↓ HCN4 ↓	Inhibition of cardiac contractility Reduced heart rate	Increased action potential duration Increased effective refractory period Inhibition of delayed and early after-depolarization	Rat Guinea pig	50, 100 and 500 μM 14.02 μmol/l 1, 50, 100 μmol/l	[196, 225–229]
**Salvianic acid A**	ICaL ↓	Antiarrhythmic effect	Decreased myocardial contractility	Rat	14.7 μM	[53]
**Tetrandrine**	BK ↓ LTCC and TTCC ↓	Antiarrhythmic effect	Decreased action potential duration Reduced Ca^2+^ influx into the cell from sarcolemma Decreased Ca^2+^ uptake into the sarcoplasmic reticulum	Rabbit Rat	7.5 and 15 μmol/l 30 μmol/l	[230–233]

**Table 4:** The effects of herbal medicine on brain and gastrointestinal ion channels

Compound	Effect on ion channels	Brain effects	GI effects	Species	Dose	Ref
**Allicin**	NTCC and P/QTCC ↓	Neuroprotective	-	Rat	20 μM	[66]
**Asarone**	ICa ↓ NaV1.2 ↓	Antiepileptic effect	-	Mouse	20 mg/kg	[73]
**Curcumin (Turmeric)**	INa and IKATP ↓	Nociceptive effect Antidepressant effect	Protection against gastric ulcer	Rat	100 mg/kg	[46, 49, 50]
**Estragole**	INa ↓	Anxiolytic effect	-	Rat	30 mg/kg	[186]
**Eugenol**	INa and ICa ↓ ICl ↓ TRPA1 ↑	Analgesic and antiepileptic effects	Antidiarrheal effect	Rat	2 mM 100 mg/kg	[104, 191, 234–236]
**Ginsenoside**	ICaL ↓ IKATP ↓	Antiepileptic effect Improvement of Alzheimer disease	Improved small intestine motility	Rat Mouse	20 μmol/l	[113, 188, 190]
**Hesperidin**	NTCC ↓	Neuroprotection Anticonvulsive effects	Improved ileus	Rat	10 and 50 mg/kg 500 mg/kg	[120, 121, 143, 237]
**Lavender**	ICaT ↓ TRPA1 ↓	Anxiolytic Antinociceptive	-	Mouse *in vitro*	30 μmol/l	[15, 17, 18]
**Magnolol**	INa, ICaL and IK ↓ TRPC4 ↓	Neuroprotection	Antidiarrhea Inhibits colonic motility	Rat Mouse	100, 300 and 500 mg/kg	[132–134]
**Melissa officinalis**	ICl ↑ ICa ↓	Anxiolytic activity	Reduced ileum contraction	Mouse	25 mg/kg	[23]
**Naringenin**	NaV1.8 ↓ BK ↑	Analgesic effect Improve motor neuron function	-	Rat *in vitro*	100 and 10 μM	[184, 238]
**Oxymartine**	IK ↓	Antinociceptive effects	-	Mouse	150 mg/kg	[154]
**Quercetin**	GABA_A-Cl- channels ↓ Acid-sensing ion channels ↓ INa ↓ ICaL and IK↑	Antiepileptic Improved cognitive deficits	Increased colon contractility	Rat Guinea pig	2 μM 30 μM	[143, 168, 239–241]
**Salvia miltiorrhiza**	Ca2+calmodulin pathway ↑		Increased ileum tonic contraction	Rat Mouse	40 μmol/ml	[39, 242, 243]
**α-(−)-Bisabolol**	INa ↓ IcaT ↓ IK ↑	Antinociceptive effects	-	Mouse	0.5, 1, 5 and 10 mM 1 μg 300 μg	[185, 244, 245]

## Abbreviations

5-HT2A: 5-hydroxytryptamine (serotonin) receptor 2A
APD: Action potential duration
BK: Large-conductance Ca^2+^-activated potassium channel
CaM: Calmodulin
CaSR: Calcium-sensing receptor
cGMP: Cyclic guanosine monophosphate
CGRP: Calcitonin gene-related peptide
CRAC: Ca^2+^ release-activated Ca^2+^ channel
DAD: Delayed after depolarization
EAD: Early after depolarization
ECG: Electrocardiogram
GABA: Gamma-aminobutyric acid
HCN: Hyperpolarization-activated cyclic nucleotide-gated channel
hERG: Human ether-à-go-go related gene
I/R: Ischemia/reperfusion
I_Ca_: Calcium current
I_CaL_: L-type calcium current
I_CaT_: T-type calcium current
I_Cl_: Chloride current
I_f_: Funny current (pacemaker current)
I_K_: Potassium current
I_K1_: Inward rectifier potassium current
I_KAch_: Potassium current via K_ACh_ channel
I_KATP_: Potassium current via K_ATP_ channel
I_Kr_: Rapid delayed-rectifier potassium current
I_Ks_: Slow delayed-rectifier potassium current
I_KM3_: M3-receptor-mediated potassium current.
I_Kur_: Ultrarapid delayed-rectifier potassium current
I_KV_: Voltage-gated potassium channel
I_Na_: Sodium current
I_NaL_: Late sodium current
I_NaT_: Transient sodium current
I_to_: Transient outward potassium current
K_ACh_: Acetylcholine-activated (muscarinic) potassium channel
K_ATP_: ATP-sensitive potassium channel
KCNH2: Potassium voltage-gated channel subfamily H member 2
KCNN3: Potassium calcium-activated channel subfamily N member 3
KCNQ: Voltage-gated Kv7 potassium channel
K_ir_: Inward-rectifier potassium channel
K_V_: Voltage-gated potassium channel
LDL: Low-density lipoprotein
L-type Ca^2+^ channel (LTCC): Large voltage-gated calcium channel
MAPK: Mitogen-activated protein kinase
mPTPs: Mitochondrial permeability transition pores
N-type Ca^2+^ channel (NTCC): Neuronal voltage-gated calcium channel
NMDA: N-methyl-D-aspartate
NR2B: N-methyl D-aspartate receptor subtype 2B
PKC: Protein kinase C
P/Q-type Ca^2+^ channel (P/QTCC): P/Q-type voltage-gated calcium channel
ROS: Reactive oxygen species
SCN5A: Sodium channel protein type 5 subunit alpha
SERCA2: Sarcoplasmic/endoplasmic reticulum calcium ATPase 2
SK: Small-conductance Ca^2+^-activated K^+^ channel
T-type Ca^2+^ channel (TTCC): Transient voltage-gated calcium channel
TRP: Transient receptor potential channel
TRPA1: Transient receptor potential cation channel A1
TRPC4: Transient receptor potential cation channel C4
